# Hydrodynamic Interaction Enhances Colonization of Sinking Nutrient Sources by Motile Microorganisms

**DOI:** 10.3389/fmicb.2019.00289

**Published:** 2019-03-12

**Authors:** Nikhil Desai, Vaseem A. Shaik, Arezoo M. Ardekani

**Affiliations:** School of Mechanical Engineering, Purdue University, West Lafayette, IN, United States

**Keywords:** marine snow, hydrodynamic interactions, chemotaxis, hydrodynamic trapping, nutrient colonization

## Abstract

In this study, we document hydrodynamics-mediated trapping of microorganisms around a moving spherical nutrient source such as a settling marine snow aggregate. There exists a range of size and excess density of the nutrient source, and motility and morphology of the microorganism under which hydrodynamic interactions enable the passive capture of approaching microorganisms onto a moving nutrient source. We simulate trajectories of chemotactic and non-chemotactic bacteria encountering a sinking marine snow particle effusing soluble nutrients. We calculate the average nutrient concentration to which the bacteria are exposed, under regimes of strong and weak hydrodynamic trapping. We find that hydrodynamic trapping can significantly amplify (by ≈40%) the nutrient exposure of bacteria, both chemotactic and non-chemotactic. The subtle interactions between hydrodynamic and chemotactic effects reveal non-trivial variations in this “hydrodynamic amplification,” as a function of relevant biophysical parameters. Our study provides a consistent description of how microorganism motility, fluid flow and nutrient distribution affect foraging by marine microbes, and the formation of biofilms on spherical nutrient sources under the influence of fluid flow.

## 1. Introduction

Chemotaxis–the directed motion of bacteria along favorable gradients in chemical concentration–is one of the primary mechanisms through which marine bacteria locate nutrition, from sources like phytoplankton, marine snow and oil drops (Stocker and Seymour, 2012). In the past, researchers have studied how chemotaxis helps in the colonization of settling particles (Kiørboe et al., 2002), and of the nutrient plumes that trail these particles (Jackson, 1989; Kiørboe and Jackson, 2001; Stocker et al., 2008). Besides, chemotaxis is also vital in following nutrient sources with inherent motility, e.g., the tracking of the motile algae *Pavlova lutheri* by the marine bacteria *Pseudoalteromonas haloplanktis* (Barbara and Mitchell, 2003). Bacteria utilize a number of strategies, like “run-and-tumble” or “run-reverse-flick,” to bias their motion to chemical cues, and find and populate nutrient-rich regions in their environment (Berg, 2004; Stocker et al., 2008; Son et al., 2016; Desai and Ardekani, 2017). These strategies are actively regulated on the level of an individual cell, via chemosensing, i.e., feedback mechanisms involving membrane receptors and intracellular signals (Eisenbach et al., 2004).

In addition to external chemical cues, microorganism locomotion is also affected by the ambient fluid flow. Microorganisms are translated and rotated by background flows and they undergo changes in their swimming motion by “interacting hydrodynamically” with their surroundings (Ramia et al., 1993; Lauga and Powers, 2009). As a bacterium swims, its appendages disturb the fluid around it, setting up a flow. The presence of bounding surfaces and/or obstacles–especially within a few body lengths from the bacterium–affects this flow, which in turn affects the motion of the bacterium itself. Thus, the fluid flow set up by a swimming bacterium is altered by nearby surfaces/interfaces, resulting in changes in the motility of the bacterium. This mechanism is called hydrodynamic interaction of the microorganism with the surface/interface. Hydrodynamic interactions have been used to successfully describe a number of non-trivial phenomena, like the circular trajectories of *E. coli* in the vicinity of plane walls (DiLuzio et al., 2005; Lauga et al., 2006) and air-fluid or fluid-fluid interfaces (Lemelle et al., 2010; Di Leonardo et al., 2011; Lopez and Lauga, 2014); the tendency of microorganisms to be attracted to and accumulate near walls (Berke et al., 2008; Li and Ardekani, 2014); the enhanced residence time of bacteria and microswimmers near plane and curved solid surfaces (Drescher et al., 2011; Takagi et al., 2014; Spagnolie et al., 2015; Jashnsaz et al., 2017). Examination of the flow fields around bacteria reveals that hydrodynamic interactions are most important at *small* cell-surface separations (Drescher et al., 2011), which suggests that they can affect the trajectories of bacteria that encounter sinking particles either by chance or through chemotaxis.

The influence of near-surface hydrodynamic interactions on foraging by marine bacteria is thus an interesting topic, which has not been considered in detail in prior studies on chemotaxis toward settling particles. Recently, Desai and Ardekani analyzed the influence of hydrodynamic interactions in the motion and distribution of chemotactic bacteria around stationary, spherical nutrient sources, and concluded that hydrodynamic interactions greatly assist in the colonization of nutrient sources (Desai and Ardekani, 2018). This significance of hydrodynamic interactions in bacterial accumulation around fixed nutrient sources motivates us to examine the combined effects of hydrodynamic interactions and chemotaxis on the distribution of marine microbes around moving (due to gravity) nutrient sources. Our study is particularly relevant in the context of microbial colonization of sinking marine snow particles, and of rising oil drops emanating from natural or anthropogenic oil spills (Atlas and Hazen, 2011). We wish to identify the factors affecting a bacterium's average nutrient exposure under these conditions. This is pivotal in determining the overall uptake rates by marine bacteria and the subsequent microbiological processes dictating bacterial populations in particular, and the marine biogeochemistry in general (Kirchman, 2008). We formulate, and solve, a mathematical model which incorporates the essential features of the mechanisms governing bacterial motion: (i) run-and-tumble chemotaxis toward a nutrient/chemoattractant emanating from a spherical, sinking nutrient source (e.g., an aggregate like marine snow), (ii) fluid flow caused by the source, and, (iii) hydrodynamic interactions caused by proximity to the nutrient source (a rigid obstacle). We emphasize here that the first response is an active motility trait of most bacteria, and the latter two are passive, i.e., driven solely by hydrodynamics. While the chemotactic response may be specific to bacterial species, the hydrodynamic effects are more generally valid. Through our analysis, we identify the effect of hydrodynamic interactions on the average nutrient exposure of marine bacteria swimming close to sinking nutrient sources. We quantify it as a function of important environmental (size of nutrient source and the diffusivity of the nutrient) and biological factors (mean run-time of the bacterium and magnitude of the force its appendages exert on the surrounding fluid).

## 2. Influence of Hydrodynamics and Chemotaxis

**Figure 1:**
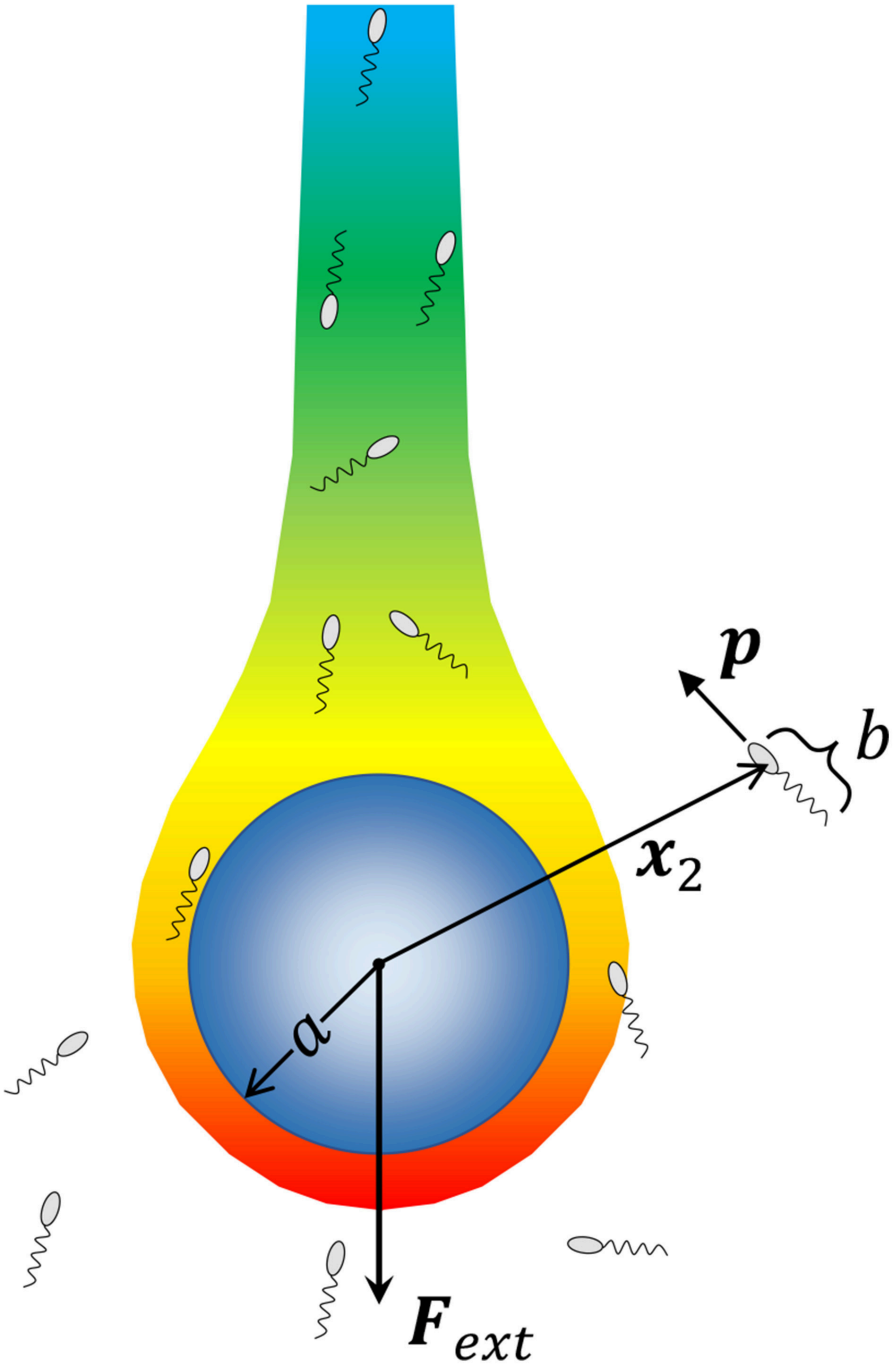
A schematic of the problem being solved. A marine snow aggregate of radius *a* sinks under the influence of an external force (gravity) **F**_*ext*_. A chemoattractant emanates from the surface of marine snow, and forms a plume “behind” the marine snow. We consider a system of *N*_*b*_ bacteria (of size *b*) that are not interacting with each other but can perform chemotaxis toward nutrient hot-spots (the concentration boundary layer and the plume around the aggregate), and interact hydrodynamically with the aggregate upon encountering it. We consider the motion of each bacterium by simulating its trajectory, i.e., the time evolution of its position with respect to the aggregate **x**_2_, and its orientation **p**, as dictated by hydrodynamic and chemotactic effects.

We consider a spherical aggregate or marine snow particle of radius *a* (shown in Figure 1), which also acts as the source of a chemoattractant/nutrient, sinking under the influence of gravity with a force **F**_*ext*_ = Δ*ρV*_*p*_**g** acting on it; where, Δρ is the excess density of marine snow [ranging from 10^−5^ g/cm^3^ to 10^−3^ g/cm^3^; (Alldredge and Gotschalk, 1988)], $V_{p}=\frac{4}{3}\pi a^{3}$ is its volume and **g** is the acceleration due to gravity. The nutrient diffusing out of the source is carried by the fluid and forms a downstream plume as shown. At a position **x**_2_ with respect to the center of this particle, lies a microorganism of size *b*. The fluid flow is affected by both the sinking particle and the microorganism. The presence of the particle is expected to affect the swimming motion of the microorganism through hydrodynamic interactions, and vice-versa. As the aggregate sinks, it encounters bacteria either because they lie in its path, or because they are attracted, via chemotaxis, to its surface. Once the bacterium-aggregate separation reduces to within a few bacterial body lengths, chemotaxis becomes less important and hydrodynamic interactions become significant. On the other hand, bacterial motion *far* from the marine snow is affected primarily by chemotaxis. We, thus, first consider the motion of bacteria due to hydrodynamics and chemotaxis separately, and then get the complete description obtained by combining the two effects.

### 2.1. Bacterium as a Force Dipole

Figure 1 shows the bacterium's location **x**_2_ with respect to the center of the marine snow, and its orientation **p**. These govern the bacterium's trajectory and evolve in time according to

(1) $$\begin{array}{l} \frac{d{\text{x}}_{2}}{dt}={\text{u}}_{HI}-{\text{U}}_{p}+V_{s}\text{p},\frac{d\text{p}}{dt}=\Omega_{HI}\times \text{p}, \end{array}$$

where **u**_*HI*_ and **Ω**_*HI*_ are the hydrodynamically induced linear and angular velocities of the bacterium, respectively; **U**_*p*_ is the velocity of the marine snow particle; and *V*_*s*_ is the swimming speed of the bacterium. Equation 1 shows that in the absence of hydrodynamic interactions–say, in an unbounded quiescent fluid–the bacterium simply swims along its instantaneous direction **p**. In order to calculate **u**_*HI*_, **Ω**_*HI*_ and **U**_*p*_, we need knowledge of the fluid flow around the bacterium. The typical size of marine bacteria ranges from 1-10 μm; for these length scales, the Reynolds number–which is the ratio of inertial forces to viscous forces–associated with fluid flow is exceedingly small. In addition, we only consider marine snow particles of diameter *d* and settling speed *U*_*p*_ such that their associated Reynolds number, *Re*_*ms*_ = ρ*U*_*p*_*d*/μ << 1, where ρ and μ are the density and viscosity of the suspending fluid, respectively. This allows us to safely neglect the effect of fluid inertia in our analysis.

**Figure 2:**
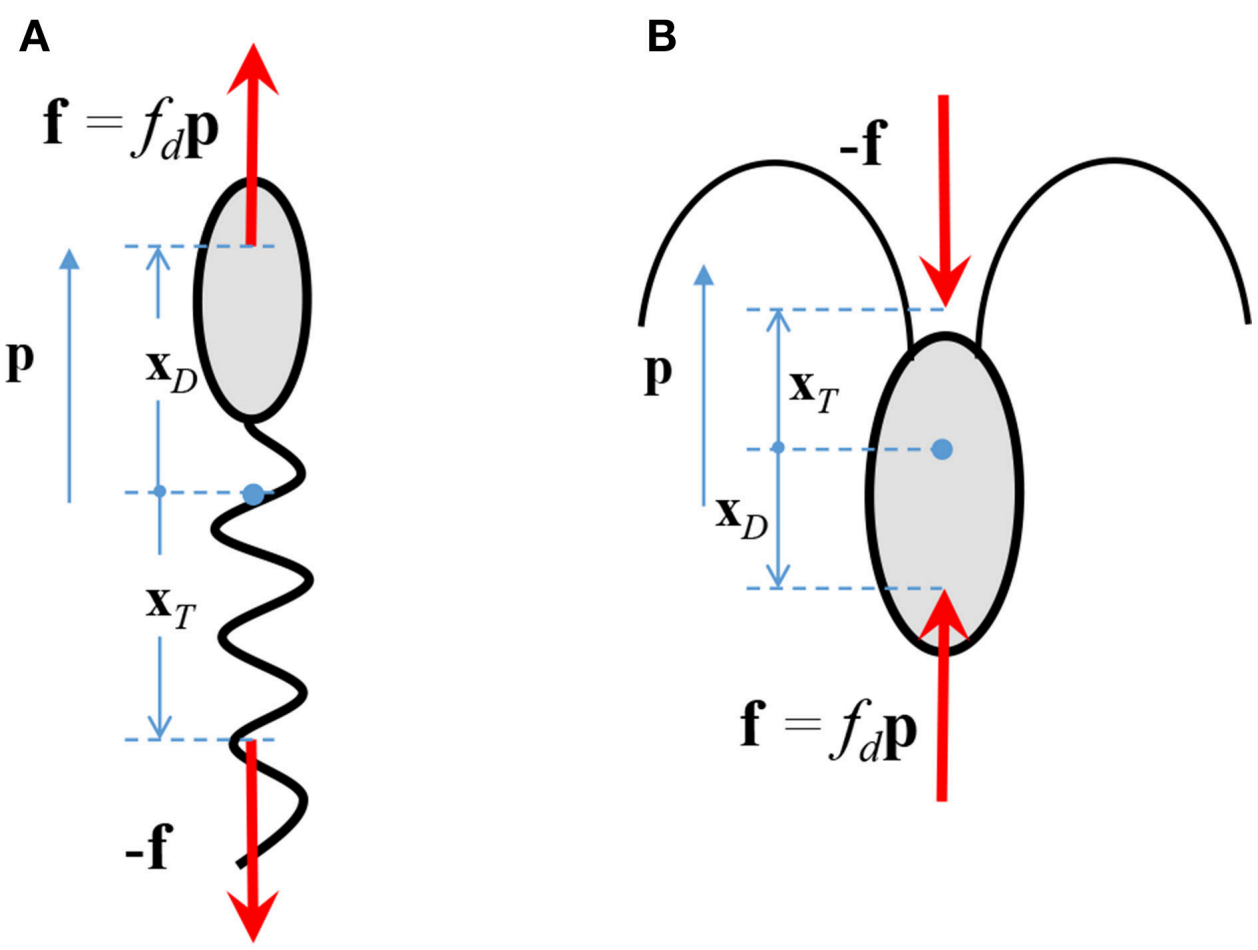
The drag, *f*_*d*_**p**, and thrust, −**f**, exerted by a **(A)** pusher, and, **(B)** puller, on the surrounding fluid. **p** is the direction in which the microorganism swims in an unbounded, quiescent fluid.

The fluid flow is governed by the equations describing the conservation of mass and momentum. We incorporate the effect of the bacterium on the fluid flow by considering it as a “force dipole,” i.e., two equal and opposite forces being exerted on the fluid by the bacterium's cell body and its flagellum/flagella (Drescher et al., 2011). The force exerted by the cell body is called the drag (say **f** = *f*_*d*_**p**), and that by the flagellum is called the thrust (-**f**). The force dipole representation arises because of the small separation between the points of application of the drag and the thrust. An important parameter in our study is the “dipole strength” of the bacterium, denoted by *F*_*D*_. Physically, it is the scalar product of the drag force exerted by the bacterium on the fluid, *f*_*d*_**p**, and the position of the point of application of the drag, with respect to the center of the bacterium, i.e., *F*_*D*_ ≈ *f*_*d*_**p**·**x**_*D*_ (see Figure 2). The magnitude of *F*_*D*_ ranges from 0.1-1 pN-μm, for a wide range of bacterial species, e.g., *Escherichia coli, Pseudomonas aeruginosa, Vibrio cholerae, Salmonella typhimurium, Vibrio alginolyticus* (Berke et al., 2008; Drescher et al., 2011; Son et al., 2013). A stronger influence of the microbe on the flow, and thus a stronger hydrodynamic interaction, occurs for larger values of *F*_*D*_. From Figure 2 and the definition of *F*_*D*_, it is clear that *F*_*D*_ > 0 for microorganisms that exert thrust near their tail (called pushers, e.g., most bacteria) and *F*_*D*_ < 0 for microorganisms that exert thrust near their head (called pullers, e.g., algae); in this work, we consider the former case. The details of the mathematical formulation are given in Appendix A, and the expressions for the various hydrodynamically induced velocities are given in Appendix B.

The dynamics described by Equation 1 is most accurate when the separation between the bacterium and the aggregate is large; as this separation reduces, the accuracy of the model deteriorates. Specifically, Berke et al. (2008) measured the distribution of *E. coli* in a suspension confined by parallel glass plates and concluded that the force dipole model ceases to be a valid approximation at distances of around 10 body lengths (i.e., at (|**x**_2_|−*a*) ≈ 10*b*) from the surface. For (|**x**_2_|−*a*) < 10*b*, the effects of finite size of the bacterium, its shape asymmetry and flagellar rotation become important and these are not captured by a force dipole. As the bacterium approaches to within touching distance from the aggregate, the force dipole model results in unrealistic effects like the penetration of the bacterium through the rigid surface of the aggregate. This can be remedied using more involved hydrodynamics but for the sake of simplicity we model the near-field interaction between the bacterium and the aggregate as a hardcore repulsion. This means that upon contact, we ensure that the bacterium doesn't penetrate into the aggregate, but moves tangentially along its surface while being free to rotate. Thus, the bacterium cannot penetrate into the aggregate but can still rotate away and escape from it after spending some time on its surface. Such steric interactions are not uncommon and have indeed been observed for a number of microorganisms in contact with rigid surfaces (Li et al., 2011; Bianchi et al., 2017).

The force dipole model has been used in the past to explain the hydrodynamic trapping of microswimmers/bacteria impinging on stationary rigid spheres (Spagnolie et al., 2015) and drops (Desai et al., 2017). This trapping phenomena has been observed experimentally as well, for both artificial micro-swimmers (Takagi et al., 2014) and for the bacterium *E. coli* and its predator *Bdellovibrio bacteriovorus* (Jashnsaz et al., 2017). In this study, we show that such trapping can also occur when a bacterium encounters a sinking sphere (see Section 3.1).

### 2.2. Bulk Nutrient Distribution and Chemotaxis

Many bacteria follow a run-and-tumble behavior, wherein their orientation, **p**, can change abruptly depending on the instantaneous rate of change of chemoattractant concentration in their vicinity. In our case, the chemoattractant concentration, *C*, satisfies the steady-state convection-diffusion equation

(2) $$\begin{array}{l} \nabla \cdot \left({\text{v}}_{St}C\right)=D_{C}\nabla^{2}C, \end{array}$$

where *D*_*C*_ is the nutrient diffusivity, and **v**_*St*_ is the flow field due to a sphere sedimenting under gravity in absence of inertial effects and in an unbounded fluid. This is a simplification, because we are not accounting for the effects of the hydrodynamic interactions (between the settling aggregate and the bacterium) on the convection-diffusion equation. In reality, hydrodynamic interactions would change the sphere's linear and angular velocities as it settles and the presence of the bacterium would disturb the fluid flow, making it different from **v**_*St*_. Thus, the fluid flow–and through it, the nutrient transport–will be affected by hydrodynamic-interaction-induced changes in the marine snow's motion, and by the bacterium-induced flow. But for the parameter range of our study (see Table 1), these changes will be negligible in comparison to the fluid flow associated with the marine snow particle's gravitational settling (see Equations 15–20 in Appendix). Therefore, we can justify the simplification made in Equation 2. We solve Equation 2 subject to the conditions that *C* = *C*_0_ at the sphere surface, and *C* → 0 at large distances away from the sphere. Note that a fixed surface concentration of the nutrient corresponds to transport limited nutrient transfer (Karp-Boss et al., 1996).

**Table 1:** List of parameters and their values used in the numerical simulations.

**Symbol**	**Description**	**Value range (units)**
**FLOW**		
μ	Viscosity of suspending fluid	0.01 (poise)
ρ	Density of suspending fluid	1.00 (g/cm^3^)
*d* = 2*a*	Diameter of marine snow	0.04–0.13 (cm)
Δρ	Excess density of marine snow	10^−4^-10^−3^ (g/cm^3^)
$U_{p}=\left(2/9\right)\Delta \rho ga^{2}/\mu$	Settling speed of marine snow	0.004−0.046 (cm/s)
*Re*_*b*_ = ρ*V*_*s*_*b*/μ	Reynolds number for bacterium	10^−5^-10^−4^
*Re*_*ms*_ = ρ*U*_*p*_*d*/μ	Reynolds number for marine snow	0.02-0.6
**BACTERIA**		
*V*_*s*_	Swimming speed	10–50 (μm/s)
*b*	Size	1–10 (μm)
α_*C*_	Chemotactic time constant	1200 (s)
τ_0_	Mean run-time	0.4–10 (s)
*D*_*r*_	Rotational diffusivity	10^−3^-10^−2^ (s^−1^)
*F*_*D*_	Bacterial dipole strength	0.1–1 (pN-μm)
$\alpha_{D}=F_{D}/\left(8\pi \mu b^{2}V_{s}\right)$	Dimensionless dipole strength	0.1–2
**NUTRIENT**		
*C*_0_	Reference concentration	25 (μM)
*K*_*D*_	Half-saturation constant of chemoreceptor	2.5–250 (μM)
*D*_*C*_	Diffusivity	4 × 10^−7^-2 × 10^−5^ (cm^2^/s)
*Sc* = ν/*D*_*C*_	Schmidt number	500–25000
*Pe* = *U*_*p*_*a*/*D*_*C*_	Péclet number	100–5000
$Pe_{b}=U_{p}a/\left(V_{s}^{2}\tau_{0}/6\right)$	bacterial Péclet number	50–40,000
$\delta_{C}\approx {\left(9\mu D_{C}/2\Delta \rho g\right)}^{1/3}$	Concentration boundary layer thickness	0.0026−0.0132 (cm)
**SIMULATION**		
Δ*t*	Dimensionless time step	10^−3^
*N*_*b*_	Number of bacteria in simulation	1000–5000
*L*_*up*_ = 5*a*	Upstream bacteria starting distance	0.1–0.38 (cm)
*R*_*disk*_ = 2*a*	Radius of disk of bacteria's initial positions	0.04–0.13 (cm)

*The parameter values have been borrowed from following references: marine snow size and density (Alldredge and Gotschalk, 1988), bacterium size, speed, dipole strengths and rotational diffusivity (Berke et al., 2008; Drescher et al., 2011; Son et al., 2013), bacterium run-times and other chemotactic parameters (Berg and Brown, 1972; Bowen et al., 1993; Kiørboe et al., 2002; Berg, 2004), nutrient diffusivities (Bowen et al., 1993; Jackson, 2012)*.

Once the concentration *C* is known, the run-and-tumble chemotaxis is implemented by prescribing the run-time τ of the bacterium as a function of *DC*/*Dt*, i.e., the instantaneous rate of change of the chemoattractant concentration as seen by the bacterium. This is done by providing a bias to the mean run-time of the bacterium in absence of chemoattractant, τ_0_, according to the relation (see Appendix C for details):

(3) $$\begin{array}{l} \tau =\left\{ \begin{array}{l} \tau_{0}\exp\left(\alpha_{C}\frac{K_{D}}{{\left(K_{D}+C\right)}^{2}}\frac{DC}{Dt}\right),\frac{DC}{Dt}>0\\ \;\tau_{0},\frac{DC}{Dt}\leq 0 \end{array}\right., \end{array}$$

where α_*C*_ is a time-constant and *K*_*D*_ is the half-saturation constant of the receptors that bind to the chemoattractant. Equation 3 shows how chemotactic bacteria can climb up nutrient gradients: by increasing their run-time whenever they swim along regions with increasing ambient nutrient concentration. One important point is that bacterial tumbling is significantly hindered when they are near solid surfaces and most tumbles are limited to the tangent plane at the surface (Molaei et al., 2014). Therefore, we restrict near-surface tumbling, and any bacterium that comes into contact with the nutrient source cannot simply tumble away and escape. Finally, we introduce stochasticity to the bacterium's orientation–stemming from flagellar imperfections and other inherent fluctuations–in between tumbles (when its orientation is governed by the second equation in Equation 1) by allowing for rotational diffusion of the orientation **p** with a diffusivity *D*_*r*_. This changes the second equation in Equation 1 to:

(4) $$\begin{array}{l} \text{p}\left(t+\Delta t\right)-\text{p}\left(t\right)=\Delta t\left(\Omega_{HI}\left(t\right)\times \text{p}\left(t\right)\right)+\sqrt{4D_{r}\Delta t}\eta_{R}\times \text{p}\left(t\right), \end{array}$$

where **η**_*R*_ is a Gaussian white noise term over the unit-sphere.

## 3. Results

The major bio-physical parameters, and their respective dimensionless representations in our study are: the bacterial dipole strength, $\alpha_{D}=F_{D}/\left(8\pi \mu b^{2}V_{s}\right)$; the mean run-time of the bacterium, $\tau^{*}=\tau_{0}V_{s}/b$; the rotational diffusivity of the bacterium, *D* = *D*_*r*_*b*/*V*_*s*_; the nutrient's molecular diffusivity *D*_*C*_, represented by the Schmidt number, *Sc* = ν/*D*_*C*_, where ν is the kinematic viscosity of the surrounding fluid (water); the radius of the settling aggregate *R* = *a*/*b*; and the excess density $K_{\Delta \rho }=2\Delta \rho gb^{2}/\left(9\mu V_{s}\right)$. Another important parameter is the Péclet number *Pe* = *U*_*p*_*a*/*D*_*C*_, which is the ratio of advective transport of the nutrient to its diffusion. The values of all these parameters are calculated by using the corresponding dimensional values listed in Table 1.

### 3.1. Hydrodynamic Trapping: With and Without Orientational Diffusion

We first discuss how hydrodynamics affects a bacterium's behavior in close proximity to sinking marine snow, in the absence of tumbling (and hence, chemotaxis), and rotational diffusion (*D*_*r*_ = 0 in Equation 4). The idea is that fluid flow caused by a bacterium, if strong enough, causes it to rotate toward a nearby rigid surface and approach it. This “hydrodynamic attraction” is balanced by hardcore repulsion, which results in the bacterium swimming tangentially to the surface. In the following discussion, the dimensionless radii (of spherical marine snow) are represented by “*R*.”

**Figure 3:**
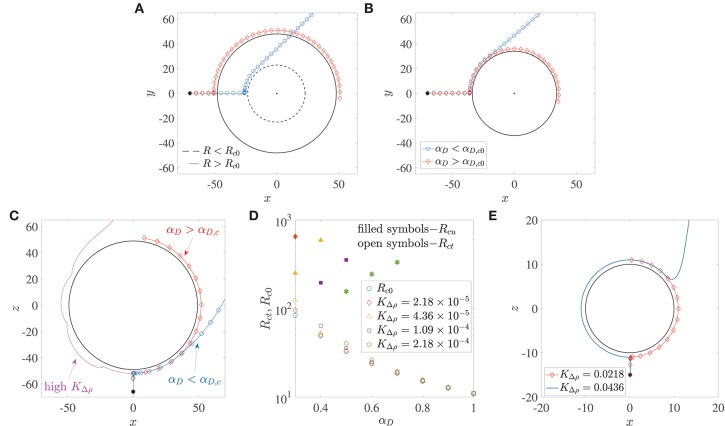
**(A)** The critical trapping radius for a stationary sphere: a bacterium gets trapped (resp. escapes) if the radius of the sphere being encountered is larger (resp. smaller) than a critical value. **(B)** Bacterium with dipole strength larger (resp. smaller) than the critical dipole strength, α_*D,c*0_ is trapped (resp. escapes) around a sphere of given radius *R*. **(C)** Trapping around a settling sphere: the trajectories are plotted in the frame of reference moving with the sphere and gravity acts along the −*z* direction; α_*D,c*_ is the critical dipole strength above which hydrodynamic trapping occurs (for a settling sphere). **(D)** Open symbols show the variation of the critical trapping radius of a sphere settling under gravity, *R*_*ct*_, with the bacterium's dipole strength, for different values of dimensionless excess density *K*_Δρ_. *R*_*c*0_ is the value of the critical trapping radius for a stationary sphere. Note that the external force acting on the particle is a function of its radius. The shape of the symbols corresponds to the *K*_Δρ_ values given in the legend. Filled symbols denote the values of the upper limit of aggregate radii *R*_*cu*_, above which the faster settling of the aggregate dominates the hydrodynamic attraction effect and thus prevents bacterium capture. These values of *R*_*cu*_ exist only for the cases shown; in all other situations, the aggregate Reynolds number becomes *O*(1) (wherein our theory becomes inapplicable) before such “rapid-settling induced escape” is seen. **(E)** An illustration of the fact that hydrodynamic capture fails to occur if the dimensionless excess density *K*_Δρ_ exceeds 4 × 10^−2^. The dimensionless bacterial dipole strength used in the simulation is α_*D*_ = 2, which is the maximum value used in this paper; and the dimensionless radius is *R* = 10, which is the minimum value considered across all our analyses. Note that trajectories for *K*_Δρ_ < 0.0218 are not shown to improve clarity, as they all overlap over the one marked by diamonds.

Microswimmers/bacteria encountering stationary spherical obstacles–like rigid spheres or liquid drops–can get trapped onto their surface due to hydrodynamic interactions, if the obstacle radius is larger than a critical radius, say *R*_*c*0_ (Spagnolie et al., 2015; Desai et al., 2017). This is shown in the trajectories in Figure 3A. The dipole strength is the same for the bacterium trajectory marked by diamonds and the one by circles; in the former, the radius of the sphere is larger than *R*_*c*0_, while in the latter, it is smaller than *R*_*c*0_. Recent experiments on the motion of *B. bacteriovorus* near beads also observed this interesting dependence of hydrodynamic trapping on sphere radius (Jashnsaz et al., 2017). A different interpretation is that spherical obstacles of a prescribed radius can (hydrodynamically) trap bacteria with dipole strengths larger than a critical value, say α_*D,c*0_. Therefore, a bacterium with dipole strength less than α_*D,c*0_, does not get trapped around a sphere (the blue trajectory marked by circles in Figure 3B), while one with dipole strength greater than α_*D,c*0_ does get trapped (the red trajectory marked by diamonds in Figure 3B). For a stationary liquid drop, the dimensionless critical trapping radius can be estimated as,

(5) $$\begin{array}{l} R_{c,drop}\approx \frac{64}{3\alpha_{D}^{2}}\frac{\lambda +1}{3\lambda +2}, \end{array}$$

where λ is the ratio of the drop's viscosity to the viscosity of its surrounding fluid. Equation 5 has been obtained from numerical calculations of the critical trapping radius for clean drops, reported in Desai et al. (2017). The critical trapping radius for a stationary rigid sphere, *R*_*c*0_, can be obtained by taking the limit λ → ∞ in Equation 5, which yields $R_{c0}\approx 64/\left(9\alpha_{D}^{2}\right)$ (Spagnolie et al., 2015). This variation is shown by the circles in Figure 3D. Alternatively, one can also evaluate the critical dipole strength for which a bacterium will trap around a rigid sphere of radius *R* by inverting the previous expression, i.e., $\alpha_{D,c0}\approx 8/\left(3R^{1/2}\right)$. If we use the diameters of marine snow particles (0.4–100 mm) as a reference, we obtain the corresponding critical dipole strength values in the range 0.05 < α_*D,c*0_ < 0.6. Measurements and calculations for *E. coli* estimate a wide range of dipole strengths, 0.01 < α_*D*_ < 2 (Darnton et al., 2007; Drescher et al., 2011), owing to heterogeneities among different cells (Chattopadhyay et al., 2006). The same is also true for other genera, like the uniflagellated marine bacterium *V. alginolyticus* (Son et al., 2013, 2016). Therefore, one can conclude that there do exist scenarios under which most motile bacteria can get hydrodynamically trapped around stationary, rigid spherical obstacles.

Does hydrodynamic trapping occur if the obstacle encountered by the bacterium is moving, instead of being fixed? To answer this, we numerically simulated (without tumbling and rotary diffusion) the dynamics of a bacterium located initially at **x**(0) = (0.1, 0, −*R*−15), and orientated along the direction opposite gravity, i.e., **p**(0) = (0, 0, 1), as shown in Figure 3C. Thus, the bacterium lies directly in the path of the sinking aggregate and eventually collides with it, after which its motion is dictated by hydrodynamic interactions with, and hardcore repulsion from the aggregate surface. In addition to the dipole strength and the sphere radius, we have a third factor that governs the bacterial dynamics when the sphere is settling under gravity: the density difference between the sphere and the ambient fluid, denoted, in dimensionless form, by *K*_Δρ_. A major difference due to gravitational settling is that if the settling speed is very large (due to large aggregate radius and/or excess density), then the bacterium cannot “keep up” with the sphere and thus cannot be trapped, as seen in the magenta trajectory in Figure 3C. This is particularly true for low dipole strengths, i.e., when the hydrodynamic interactions between the bacterium and the sphere are weak. But there exists a range of sizes (0.2 mm < *a* < 0.65 mm) and excess densities (10^−4^ g/cm^3^ < Δρ < 10^−3^ g/cm^3^) of marine snow for which hydrodynamic trapping occurs (Alldredge and Gotschalk, 1988), specifically if the bacterium's dipole strengths are large. Figure 3D shows that for excess density values that are representative of our system, substantial differences between the critical trapping radii of the stationary (*R*_*c*0_) and the translating (*R*_*ct*_) case occur only for small bacterial dipole strengths. In this regime (α_*D*_ < 0.6 in Figure 3D), the critical trapping radius for the case of a sinking aggregate increases as the excess density of the aggregate increases. However, there is no *R*_*ct*_ shown corresponding to α_*D*_ = 0.3 for $K_{\Delta \rho }=1.09\times 10^{-4}$, and corresponding to α_*D*_ = 0.3, 0.4 for $K_{\Delta \rho }=2.18\times 10^{-4}$. For these parameter values, trapping does occur at larger values of *R*, but the Reynolds number of the aggregate corresponding to these large values is ~*O*(1), and so our theory is not valid in those regimes. It is interesting that even though larger spheres settle faster, they also have a greater “hydrodynamic pull” on a bacterium with large enough dipole strength. Intuitively, one would expect larger spheres/aggregates to be less effective hydrodynamic traps as they settle faster and so an approaching bacterium might not be able to keep up with the settling sphere. But for the range of excess densities considered (10^−4^ g/cm^3^ to 10^−3^ g/cm^3^), our analysis shows that an increase in aggregate radius also strengthens the hydrodynamic interaction between the aggregate and the bacterium. This enables larger aggregates to act as more effective traps for nearby bacteria. In this way, a sphere of radius less than the critical trapping radius sinks slowly but still doesn't trap an approaching bacterium (as the hydrodynamic interaction effects are weak), while one with radius larger than the critical trapping radius sinks more rapidly yet it manages to trap oncoming bacteria with large enough dipole strength (due to stronger hydrodynamic interactions). But this effect of larger aggregate radii being more conducive to trapping might not extend indefinitely, as eventually the aggregate Reynolds number will become ~ *O*(1), and the ideas presented here will become inapplicable. In the low Reynolds number regime discussed here, there is an upper limit of aggregate radii–albeit in a few cases–above which bacteria with smaller dipole strengths fail to remain hydrodynamically bound to the aggregate. This upper limit, *R*_*cu*_, is shown whenever it exists, via filled symbols in Figure 3D. This upper limit of aggregate radius exists because the hydrodynamic trapping effect competes with the settling rate of the sphere, and there does exist some threshold settling speed above which the sphere's fast settling precludes hydrodynamic capture altogether. In accordance with this idea, we also see that if the excess density is too high ($K_{\Delta \rho }>4\times 10^{-2}$) then hydrodynamic trapping does not occur for realistic values of the bacterium dipole strength and marine snow radius. This upper limit of *K*_Δρ_ was computed by simulating the encounter of a bacterium of dipole strength α_*D*_ = 2 (which is the maximum value used in our work), with an aggregate of radius *R* = 10 (which is the minimum value used in our work). As the value of *K*_Δρ_ was increased from 2.18 × 10^−5^, the bacterium got trapped until $K_{\Delta \rho }=4.36\times 10^{-2}$ (see Figure 3E). Thus, when $K_{\Delta \rho }\geq 4.36\times 10^{-2}$, even the bacterium with highest dipole strength considered will fail to get trapped to any aggregate that we have considered in this study. An increase in the sphere size at this value of the excess density also does not favor trapping, because it further increases the sphere's settling speed, without yielding greater advantages for hydrodynamics based trapping. Since even intra-species bacterial dipole strengths can span a wide range–owing to their dependence on cell size, shape and swimming speed, one can expect a multitude of behaviors in reality. The conclusion therefore is that hydrodynamic trapping around a sinking sphere depends acutely on the sphere's excess density and the bacterium's dipole strength.

**Figure 4:**
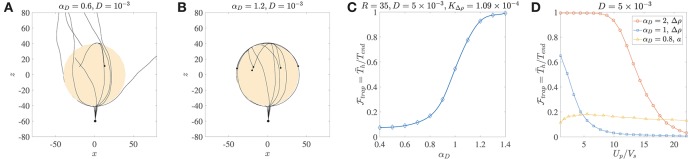
**(A)** Ten trajectories of bacteria/micro-swimmers (dipole strength α_*D*_ = 0.6, dimensionless rotary diffusivity *D* = 10^−3^) encountering a sinking sphere of radius *R* = 40, $K_{\Delta \rho }=4.36\times 10^{-4}$. Note that even though the sphere radius is larger than the corresponding critical trapping radius (*R*_*ct*_ = 28.3), only a few swimmers are trapped if their rotary diffusion is high. The swimmers spend different amounts of time on the sphere surface, before escaping from it. **(B)** Ten trajectories of micro-swimmers with a higher dipole strength (α_*D*_ = 1.2) than case **(A)**, but same dimensionless rotary diffusivity (*D* = 10^−3^) encountering the same sinking sphere as in **(A)**. In this case, all the swimmers stay hydrodynamically trapped upon encountering the sphere, because of their much larger dipole strengths. **(C)** The trapping fraction $F_{trap}$ as a function of the dipole strength for parameter values given in the title, with *T*_*end*_ = 500. Clearly, $F_{trap}\to 1$ if the swimmer's dipole strength is large enough. **(D)** The trapping fraction as a function of the aggregate's settling speed (*U*_*p*_) normalized by the bacterium's swimming speed (*V*_*s*_). The legend contains values of the dipole strength, along with the parameter that was varied (to vary *U*_*p*_) in each case. In the plots marked by circles and squares, *T*_*end*_ = 500; *U*_*p*_ is changed by changing the excess density (Δρ) of the aggregate from 10^−4^ g/cm^3^ to 2 × 10^−3^ g/cm^3^, and aggregate radius is fixed at *a* = 45 μm. In the plots marked by triangles, *T*_*end*_ = 3000; *U*_*p*_ is changed by changing the aggregate radius from 20 μm to 450 μm, and excess density is fixed at 5 × 10^−4^ g/cm^3^. The bacterium size is *b* = 1 μm.

The above behavior is deterministic because we have neglected the bacterium's rotational diffusivity. In the deterministic case, a bacterium encountering a sinking obstacle is either trapped, or it escapes, depending on the sphere's radius, its excess density and the bacterium's dipole strength. But stochasticity is introduced because of noise/rotational diffusion in the bacterium's orientation, quantified by the dimensionless parameter *D* = *D*_*r*_*b*/*V*_*s*_, where *D*_*r*_ is the rotary diffusivity of the bacterium. If the bacterium's rotary diffusivity is large, then its escape is possible even if the radius of the spherical obstacle is larger than the critical values shown in Figure 3 (see Figure 4A). A large enough rotary diffusion may overpower the fluid-flow induced rotation of the bacterium toward the aggregate. This can cause it to reorient away from the surface of the nutrient source, and simply swim away to escape the hydrodynamic entrapment (see Spagnolie et al., 2015; Desai and Ardekani, 2018 for details). In presence of noise, the bacterium's interaction with the aggregate is no longer binary (i.e., either trap or escape), and the time a bacterium spends at the surface of the aggregate is a random variable which we call the “trapping time.”

The distribution of the trapping time, *T*_*h*_, depends on the size and excess density of the aggregate, and the dipole strength and rotational diffusivity of the bacterium. We use it to quantify the trapping fraction, $F_{trap}$, defined as the mean trapping time in a simulation of 1000 bacteria divided by the total simulation time, i.e., $F_{trap}={\bar{T}}_{h}/T_{end}$, where ${\bar{T}}_{h}$ is the mean over all trial simulations. Figure 4C shows that for typical values of the bacterial rotational diffusivity, hydrodynamic trapping is still very likely for α_*D*_>1; thus suggesting that the trapping mechanism is quite robust to noise (see also Figure 4B). As an example of bacteria with α_*D*_>1, consider *E. coli* or *V. alginolyticus* cells (in water) of size *b* ≈ 1 μm, swimming speed 22 ± 5 μm/s, and dipole strength *F*_*D*_ ≈ 0.4−0.6 pN-μm (Drescher et al., 2011; Son et al., 2013). In Figure 4D, we plot the trapping fraction as a function of *U*_*p*_/*V*_*s*_, i.e., the aggregate's sinking speed in an unbounded fluid, divided by the bacterium's swimming speed. The aggregate's speed depends on its excess density Δρ and radius *a*; and we have plotted $F_{trap}$ for the cases where Δρ or *a* is varied independently. An increase in Δρ increases the settling speed and weakens the hydrodynamic attraction effect, therefore $F_{trap}$ reduces monotonically with *U*_*p*_/*V*_*s*_. Noticeably, if the bacterium's dipole strength is large then hydrodynamic trapping is quite likely ($F_{trap}\approx 0.95$) even when *U*_*p*_/*V*_*s*_ ≈ 10. The nature of $F_{trap}$ vs. *a* is non-monotonic because an increased aggregate radius affects both the settling speed and the hydrodynamic interactions (as seen in Figure 3). Higher settling speeds on account of larger aggregate radii do not necessarily diminish hydrodynamic capture, reflected in the gradual initial increase of $F_{trap}$ as *U*_*p*_/*V*_*s*_ increases from ≈ 1 to ≈ 6. This was also apparent in the results shown in Figure 3D, and is attributed to the fact that hydrodynamic attraction is enhanced for larger radii. But this enhancement does not last indefinitely and as the aggregate's radius increases further (i.e., when *U*_*p*_/*V*_*s*_ > 6 in Figure 4D) we begin to see a decline in the trapping fraction. This is because hydrodynamic attraction is now being overpowered by the more rapid settling of the aggregate and the rotary diffusion, making it exceedingly difficult for the bacterium to be retained on the surface of the sinking aggregate.

It is to be noted that the trapping behavior discussed above depends on whether a “direct encounter” takes place between the bacterium and the sphere. The most common way such an encounter may happen is if the bacterium lies in the swept volume below a settling marine snow particle. Another possibility is chemotaxis toward the surface of the nutrient-effusing marine snow, although this will depend strongly on the relative speeds and on the strength of chemotaxis. Irrespective of the mechanism of the initial contact, hydrodynamic interaction plays a crucial role in enhancing the nutrient exposure of marine bacteria. In the subsequent sections, we demonstrate this enhancement and explain the factors affecting it.

### 3.2. Average Nutrient Exposure and the Hydrodynamic Amplification

In this section, we combine the hydrodynamic and chemotactic effects described in Sections 2.1 and 2.2 to simulate the trajectories of marine bacteria encountering a sinking nutrient source. The complete details of the simulation methodology are given in Appendix C. In case of a stationary source, the nutrient concentration is spatially symmetric and the solution to Equation 2 (with **v** = 0) is just *C*/*C*_0_ = *a*/*r*; thus there is abundant nutrient availability all around the source. This changes as the source settles under gravity because the nutrient which diffuses out of its surface gets convected downstream as a plume (see **Figure 6A**). The width of this plume can be thought of as a measure of the spatial “nutrient availability,” with wider plumes being more amenable to location and population by bacteria via chemotaxis. An equivalent metric is the concentration boundary layer thickness, denoted by δ_*C*_. It is defined roughly as the (small) radial distance from the source, transverse to the settling direction, within which the nutrient concentration *C* drops from *C*_0_ to within 1% of *C*_0_. This boundary layer thickness depends on the nutrient's Péclet number as $\delta_{C}\sim aPe^{-1/3}$, for *Pe* >> 1, and *Re*_*ms*_ << 1 (Leal, 2007). **Figure 6** shows how the boundary layer thickness reduces as *Pe* increases due to reducing nutrient diffusivity.

**Figure 5:**
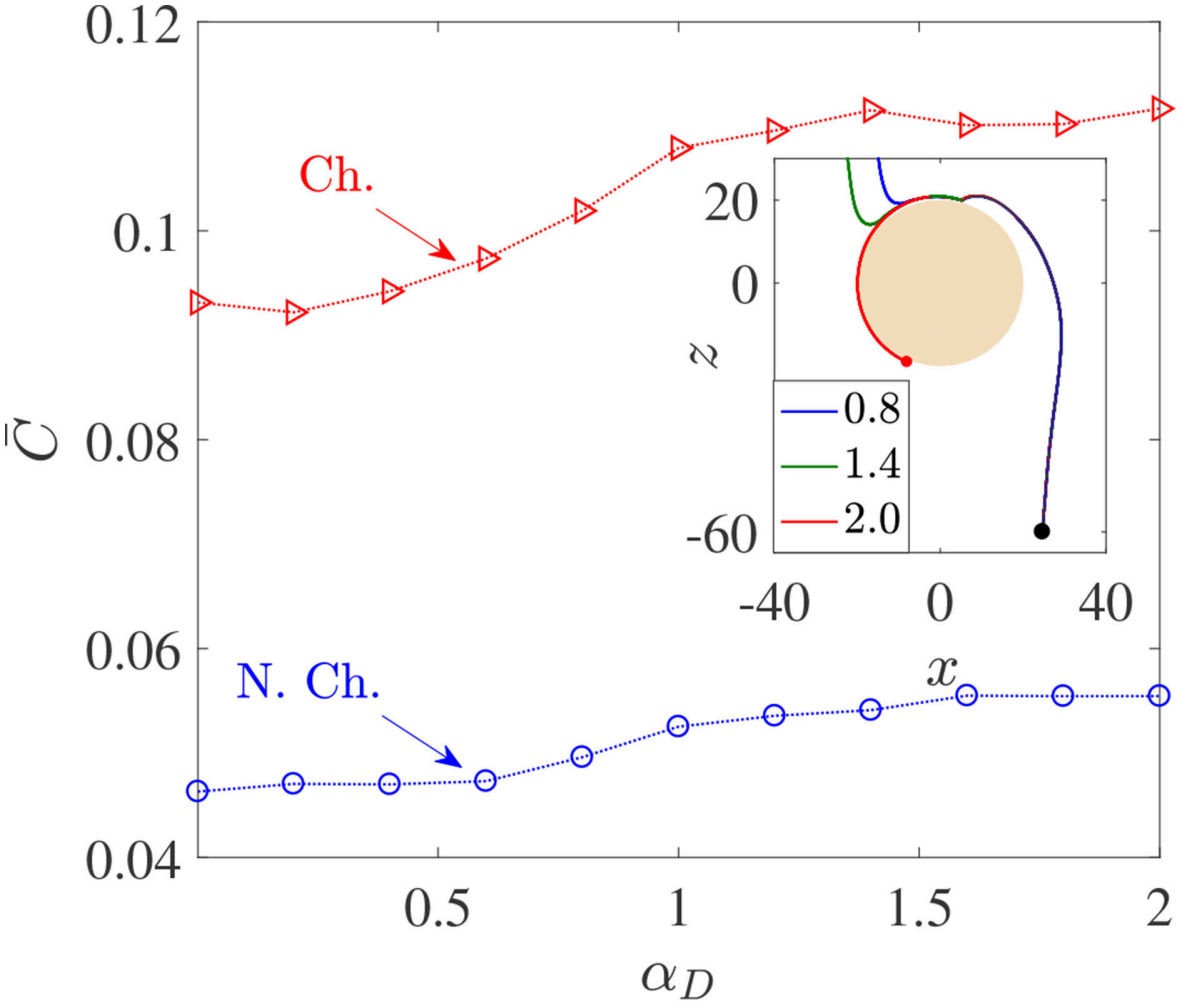
Variation in average nutrient exposure, $\bar{C}$, as a function of the dimensionless bacterial dipole strength α_*D*_, for chemotactic (Ch.) and non-chemotactic (N.Ch.) bacteria. The other parameters are: *R* = 45, *K*_Δρ_ = 0.0109, *Sc* = 1000, τ^*^ = 1. Inset: The trajectories of three chemotactic bacteria with different α_*D*_ values (these are given in the legend). It can be seen that all three trajectories begin just outside the aggregate's swept volume but are able to “chemotax” onto the surface. The amount of time each bacterium spends on the surface of the source depends on their dipole strengths.

**Figure 6:**
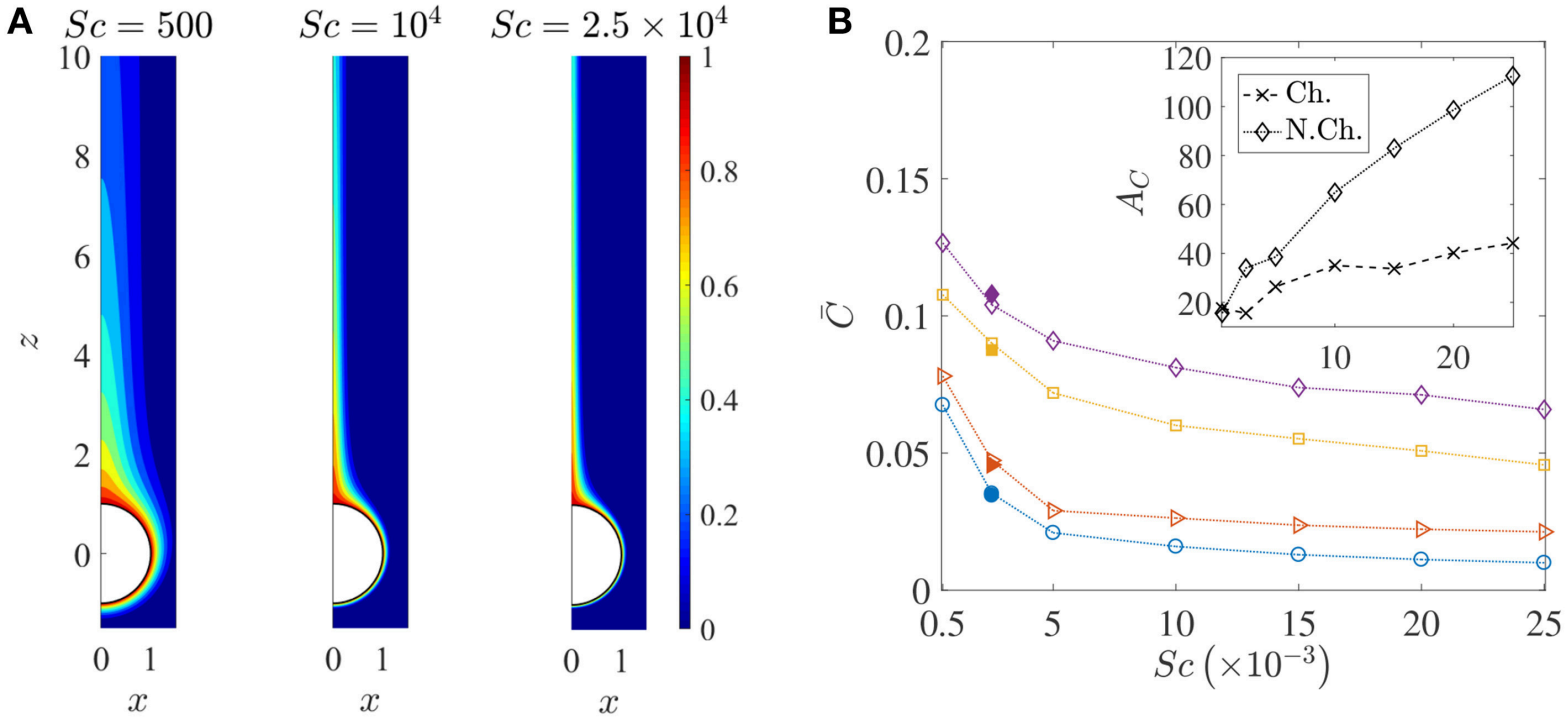
**(A)** Spatial variation of the nutrient's (normalized) concentration around the sinking sphere. The thickness of the concentration boundary layer, δ_*C*_, reduces as the nutrient diffusivity reduces. The corresponding values of the Péclet number are 100, 2000, 5000. In this figure, the distances are normalized by the radius of the sinking aggregate. **(B)** The variation in the average nutrient exposure, $\bar{C}$, for chemotactic and non-chemotactic bacteria, with strong and weak hydrodynamic interactions, as a function of the Schmidt number. The legends in the main figure are as follows: ◇−chemotactic, α_*D*_ = 2; □−chemotactic, α_*D*_ = 0.1; ⊳−non-chemotactic, α_*D*_ = 2; ○−non-chemotactic, α_*D*_ = 0.1. The filled symbols (for *Sc* = 2500) correspond to simulation results with *N*_*b*_ = 5000 bacteria, while the open symbols correspond to simulation results with *N*_*b*_ = 1000 bacteria. Inset: The hydrodynamic amplification, *A*_*C*_, as a function of *Sc*, comparing separately the percentage increase in nutrient exposures for chemotactic and non-chemotactic bacteria (recall the definition of *A*_*C*_ from Equation 7). The other parameters are: *R* = 45, *K*_Δρ_ = 0.0109, τ^*^ = 1.

In our simulations, as the bacteria swim past the sinking source they either (i) encounter it (via chemotaxis or otherwise), (ii) enter the boundary layer but do not come into contact with the source, (iii) swim past the source but into the plume, or, (iv) just swim past the source with minimal hydrodynamic interaction and/or nutrient exposure. The behaviors are shown in Figures 5, **9**. Chemotaxis is key for cases (i) through (iii), while hydrodynamics is most important for the case (i). Our aim is to compute the bacteria's nutrient exposure as a function of various bio-physical parameters governing the problem's hydrodynamic and chemotactic influences. Toward this, we define the average nutrient exposure as:

(6) $$\begin{array}{l} \bar{C}=\frac{\overset{N_{b}}{\underset{i=1}{\sum }}\overset{T_{end}}{\underset{0}{\int }}C_{i}\left(t\right)dt/\left(C_{0}T_{end}\right)}{N_{b}}, \end{array}$$

where *C*_*i*_(*t*) is the nutrient history of the *i*-th bacterium and *T*_*end*_ is the simulation-time for it. We use the subscripts *Ch*. and *N*.*Ch*. to refer to the average nutrient concentrations for chemotactic and non-chemotactic bacteria, respectively. We simulate the system for four different “bacteria types”: either chemotactic or non-chemotactic, with either high or low dipole strengths (see the legend description of Figure 6B). Next, we define a term called the “hydrodynamic amplification,” i.e., the (possible) increase in the nutrient exposure, attributable to hydrodynamic interactions:

(7) $$\begin{array}{l} A_{C}=\frac{\left({\bar{C}}_{H}-{\bar{C}}_{L}\right)}{{\bar{C}}_{L}}\times 100, \end{array}$$

where the sub-scripts *H* and *L* refer, respectively, to the cases in which the hydrodynamic interactions are high/strong and low/weak. The varying strengths of these hydrodynamic interactions could be due to the aggregate's size and excess density, or the bacterium's motility characteristics represented via the dipole strength α_*D*_. In our study we focus on the amplification stemming from the dipole strength, and use α_*D*_ = 2 (resp. α_*D*_ = 0.1) for the case of strong (resp. weak) hydrodynamic interactions. Thus, the value of *A*_*C*_ will be indicative of whether hydrodynamics is of significant nutritional benefit or not. We postulate that near-surface hydrodynamic interaction significantly increases nutrient exposure as it affects colonization of moving nutrient sources, particularly by the bacteria having large dipole strengths. As explained in Section 3.1, this is because strong hydrodynamic attraction results in the bacteria getting trapped on the surface of the nutrient source, instead of just glancing the surface and getting swept away (recall the trajectories in Figure 3C).

Figure 5 shows that there is indeed a gentle increase in the value of $\bar{C}$ as the (dimensionless) dipole strength, α_*D*_, increases from 0.1 to 2, both for chemotactic (${\bar{C}}_{Ch.}$) and non-chemotactic (${\bar{C}}_{N.Ch.}$) bacteria. Naturally, chemotaxis enables the former to have more than two-folds higher average nutrient exposure, as also remarked by Jackson (1989). It is the reinforcing effect of the hydrodynamic interactions with an increase in the dimensionless dipole strength which is of major significance. The increment is not exactly monotonic and most of it occurs over the range 0.5 < α_*D*_ < 1.5. There are upper (resp. lower) limits beyond which an increased (resp. reduced) dipole strength doesn't yield proportionate increments (resp. reductions) in $\bar{C}$. The reason simply is that for very low dipole strengths, any bacterium encountering the source doesn't spend enough time swimming along its surface. In fact, the time a bacterium spends on the source increases as the dimensionless dipole strength increases, to an upper limit after which the bacterium gets trapped and does not escape. This can be seen in the inset of Figure 5: the dipole strength is highest for the red (trapped) trajectory, followed by that for the green and then the blue trajectory. Clearly, the time spent in contact with the source–and thus in a region of maximum nutrient concentration–is directly related to the dipole strength. Therefore, α_*D*_ < 0.5 (resp. α_*D*_>1.5) represents very weak (resp. strong) hydrodynamic interactions, leading to negligible changes in $\bar{C}$ in those regimes. In the former case, the bacterial residence time (on aggregate-surface) is not long enough, and in the latter case there is a saturation due to sufficiently strong hydrodynamic interactions. The intermediate region reflects the non-trivial balance between deterministic trapping and stochasticity, as explained in Section 3.1.

We saw that hydrodynamic interactions indeed enhance the average nutrient exposure for both chemotactic and non-chemotactic bacteria. More precisely, the hydrodynamic amplification, *A*_*C*_, as defined in Equation 7 is ≈ 20% for both chemotactic and non-chemotactic bacteria, when comparing the $\bar{C}$ values in Figure 5 for the weakest and the strongest hydrodynamic interactions. Next, we analyze the dependence of the average nutrient exposure on the nutrient's diffusivity, quantified in our simulations by the Schmidt number, *Sc*. Note that lower values of nutrient diffusivity mean higher values of *Sc*.

Figure 6A shows how the nutrient is restricted to a narrower region around the source as its diffusivity decreases, and the effect of this is seen in the reduction of the average nutrient exposure with increasing values of the Schmidt number for all combinations of chemotactic/non-chemotactic bacteria with strong/weak hydrodynamic interactions (see Figure 6B). This is to be expected though, as in general, a reduction in nutrient diffusivity will reduce the number of bacteria that encounter the source due to chemotaxis, and will also reduce the likelihood of most bacteria in the bulk–chemotactic or otherwise–to populate the nutrient-rich plume. The more interesting aspect can be seen in the inset, wherein stronger hydrodynamic interactions become much more beneficial as the nutrient availability reduces; particularly for the non-chemotactic bacteria wherein they experience more than double the nutrient exposure if hydrodynamic interactions are strong enough. The reason is that hydrodynamic interactions, being a purely passive phenomenon, do not depend on the nature of the nutrient that bacteria seek. They are influenced only by the morphology of the bacteria (via dipole strength, rotational diffusivity) and the size of the sinking nutrient source. Non-chemotactic bacteria can experience nutrient-rich regions in the bulk only by chance. If they do encounter the nutrient source, then the bacteria with low dipole strengths spend very little time on the aggregate surface. In essence, non-chemotactic bacteria with low dipole strengths have no way to maximize their nutrient exposure. Non-chemotactic bacteria with high dipole strengths on the other hand, get trapped onto the nutrient source whenever they encounter it, which greatly benefits them, particularly when nutrients are scarce (high values of *Sc*). The same explanation applies to chemotactic bacteria as well, but the amplification is not as high. This is because chemotaxis, if reasonably strong, enables chemotactic bacteria with lower dipole strengths to remain in the proximity of the source or in the nutrient-rich plume behind the source. This somewhat reduces their nutrient deficit as compared to their counterparts with higher dipole strengths.

**Figure 7:**
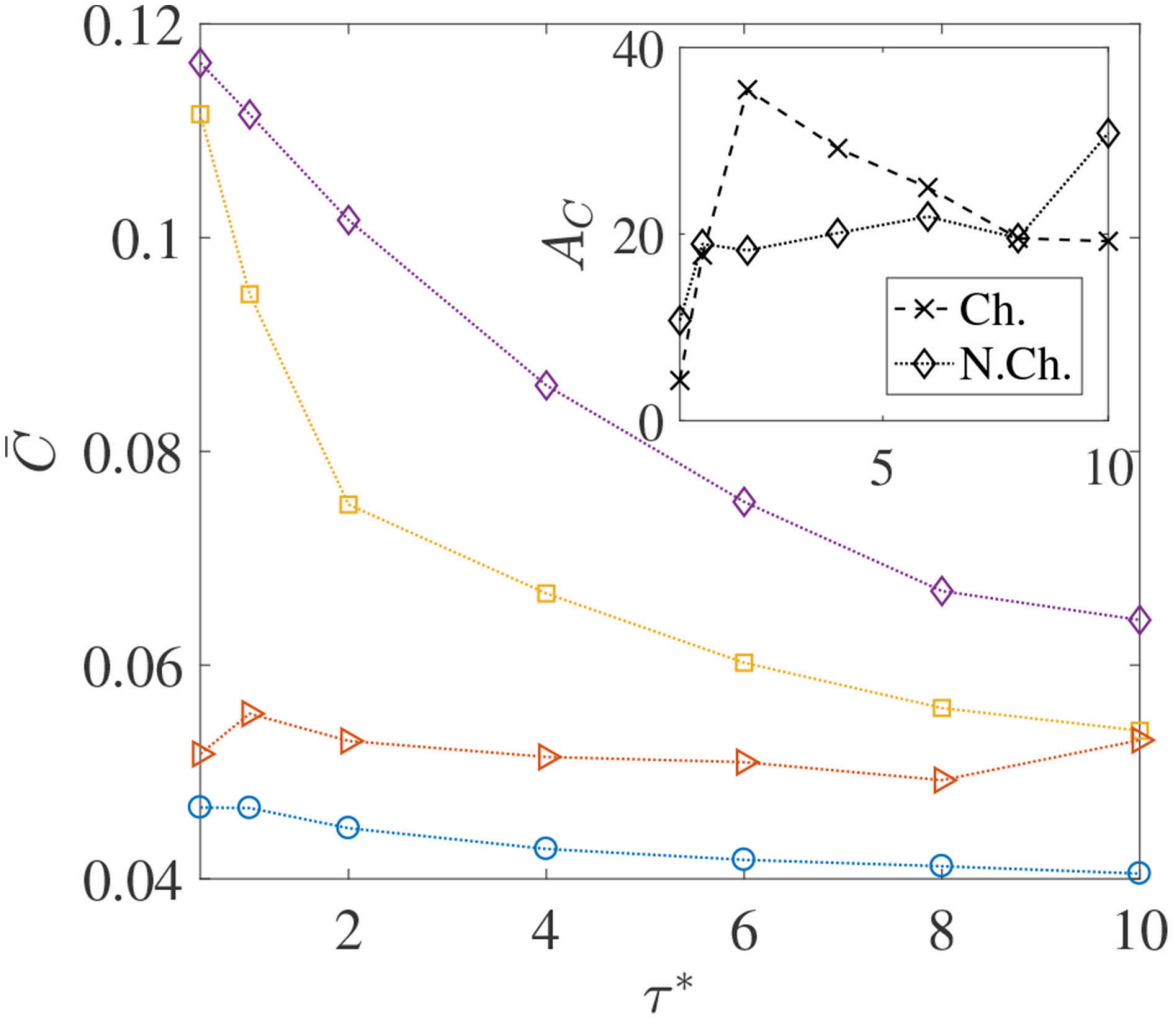
The variation in the average nutrient exposure, $\bar{C}$, for chemotactic and non-chemotactic bacteria, with strong and weak hydrodynamic interactions, as a function of the (dimensionless) mean run-time τ^*^. The legends in the main figure are as follows: ◇−chemotactic, α_*D*_ = 2; □−chemotactic, α_*D*_ = 0.1; ⊳−non-chemotactic, α_*D*_ = 2; ○−non-chemotactic, α_*D*_ = 0.1. Inset: The hydrodynamic amplification, *A*_*C*_, as a function of τ^*^. The other parameters are: *R* = 45, *K*_Δρ_ = 0.0109, *Sc* = 1000.

We saw through Figure 6 that thicker concentration boundary layers around sinking aggregates favor foraging. This was because bacteria could easily enter the boundary layer and increase nutrient availability. This idea can be succinctly explained by considering the system of *N*_*b*_ non-interacting bacteria as a continuum with “self-diffusion coefficient” *D*_*b*_, which scales as $\sim V_{s}^{2}\tau_{0}$, and then defining a bacterial Péclet number *Pe*_*b*_ = *U*_*p*_*a*/*D*_*b*_ (see Chen et al., 1998; Bearon, 2007 for details and applicability of such a simplification). Now, because the “bacterial boundary layer” around the nutrient source scales as $\delta_{B}\sim aPe_{b}^{-1/3}$, and the nutrient boundary layer scales as $\delta_{C}\sim aPe^{-1/3}$, the ratio $\delta_{B}/\delta_{C}={\left(Pe/Pe_{b}\right)}^{1/3}$ decides whether bacteria can effectively colonize the nutrient hot-spots. In the present study, *Pe*_*b*_ ranges from 50 to 40,000. As long as *Pe*_*b*_>*Pe*, the bacteria can form a boundary layer thinner than the nutrient boundary layer, i.e., δ_*B*_ < δ_*C*_ and so chemotaxis will be profitable. As *Pe*_*b*_ reduces, so does the bacterial accumulation around the nutrient source and thus the average nutrient exposure should decline. This concept is borne out in our simulations too–especially for the chemotactic bacteria–as the plots of $\bar{C}$ vs. the dimensionless mean run-time τ^*^ show, in Figure 7. For non-chemotactic bacteria with α_*D*_ = 2, the variation is fairly non-monotonic because there is no “directionality” to their motion. Their nutrient exposure depends mostly on their direct encounter with the aggregate. Bearon (2007) predicted that the encounter rate *E*_*r*_ of non-chemotactic bacteria with the aggregate varies non-monotonically with τ_0_ via a scaling $E_{r}\sim \tau_{0}^{2/3}I\left(\tau_{0}\right)$, where *I*(τ_0_) is a complicated function characterizing the effect of fluid-flow-induced rotation on the bacterial trajectories. The function *I*(τ_0_) decreases as τ_0_ increases, thus leading to non-monotonic variation of the encounter rate, *E*_*r*_; based on the results for smaller τ_0_ values, this is also reflected in our simulations. The inset in Figure 7 shows that the hydrodynamic amplification, for chemotactic bacteria, varies non-monotonically as τ^*^ increases and a maximum of *A*_*C, max*_ ≈ 35% is reached at $\tau_{0,opt}^{*}\approx 2$. A physical interpretation is that chemotaxis is too strong for τ_0_ < τ_0, *opt*_, and thus even weak hydrodynamic interactions cannot prevent the bacteria with the smallest mean run-times from either locating nutrient-rich regions in the bulk (the concentration boundary layer), or from staying close enough to the sinking nutrient source. As a result, the amplification is only ≈ 5% for the lowest self-diffusion coefficient of bacteria ($D_{b,min.}=5\times 10^{-7}$ cm^2^/s) being considered in our study. On the other hand, for the larger mean run-times of τ_0_ > τ_0, *opt*_, the reduction in the hydrodynamic amplification can be explained by the weaker chemotaxis leading to lesser colonization of the aggregate surface by bacteria with high dipole strengths. Due to this, hydrodynamics is unable to affect the nutrient exposure as severely as it does for τ_0_ < τ_0, *opt*_, resulting in obtained reduction in the values of *A*_*C*_.

**Figure 8:**
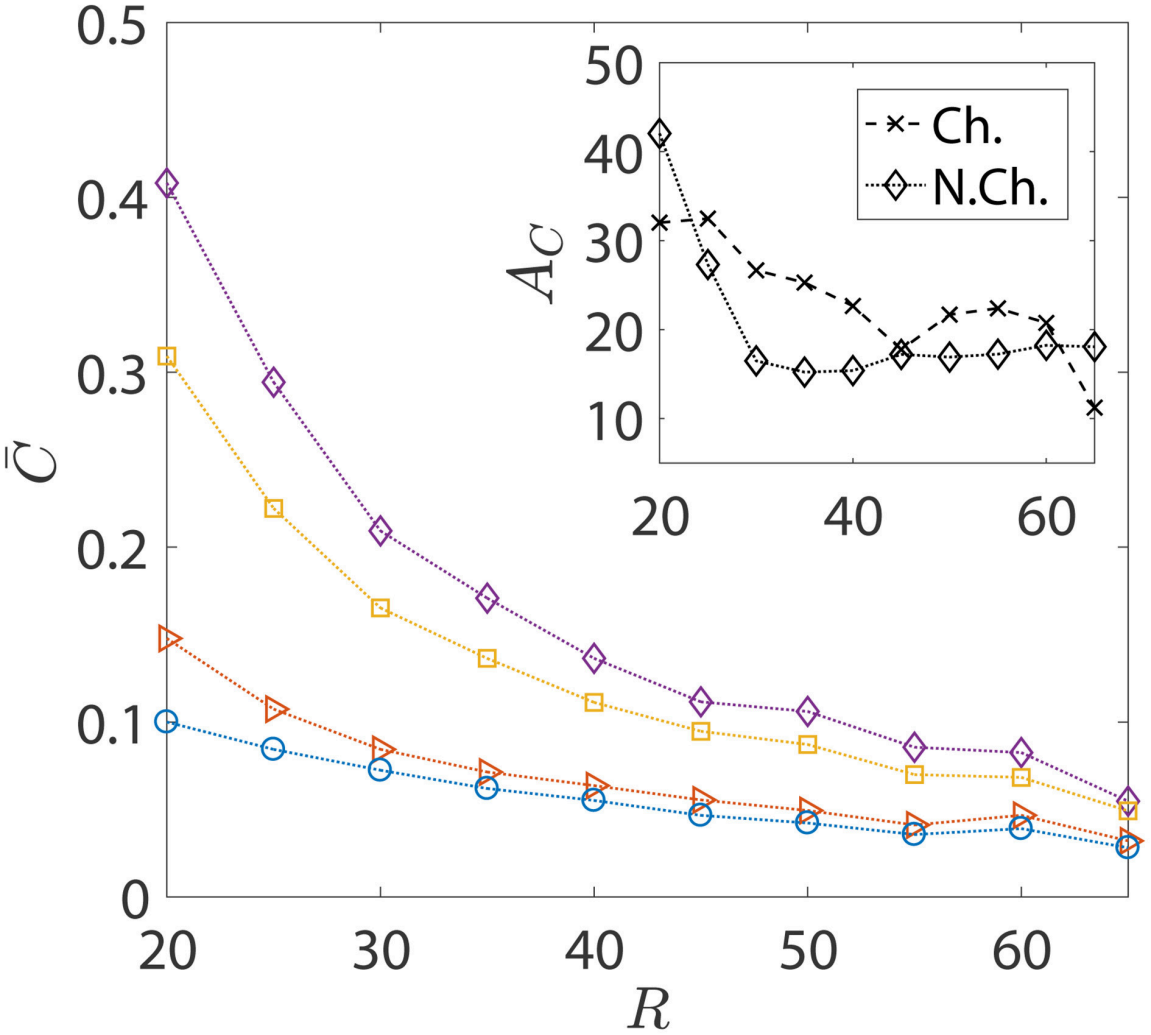
The variation in the average nutrient exposure, $\bar{C}$, for chemotactic and non-chemotactic bacteria, with strong and weak hydrodynamic interactions, as a function of the (dimensionless) radius of the marine snow particle *R*. The legends in the main figure are as follows: ◇−chemotactic, α_*D*_ = 2; □−chemotactic, α_*D*_ = 0.1; ⊳−non-chemotactic, α_*D*_ = 2; ○−non-chemotactic, α_*D*_ = 0.1. Inset: The hydrodynamic amplification, *A*_*C*_, as a function of *R*. The other parameters are: *K*_Δρ_ = 0.0109, τ^*^ = 1, *Sc* = 1000.

In the foregoing discussions, the size of the sinking aggregate, and thus its sinking speed, was fixed. The effect of hydrodynamic interactions entered the discussion via the different dipole strengths of the bacteria, with trapping (resp. escaping) being favored by high (resp. low) dipole strengths. Figure 8 details the changes in the nutrient exposure and the corresponding hydrodynamic amplifications as a function of the aggregate size. A change in the aggregate size has two implications: the first is that larger aggregates sink more rapidly and thus it becomes difficult for chemotactic bacteria to “catch up” and get trapped onto them. Therefore, even though higher aggregate radius is suitable for hydrodynamic trapping (Section 3.1), it doesn't help because of the large initial separations between the bacteria and the aggregate in our simulations. On the other hand, smaller aggregates sink slowly, giving plenty of time for chemotactic bacteria with high dipole strengths to locate the nutrient source and get trapped onto it. This is why the hydrodynamic amplification reduces, on average, as the size of the aggregate increases: the significance of hydrodynamic interactions diminishes and so does the difference between the behaviors of bacteria based on their dipole strengths.

### 3.3. Motile, Non-chemotactic Bacteria vs. Non-motile Bacteria

**Figure 9:**
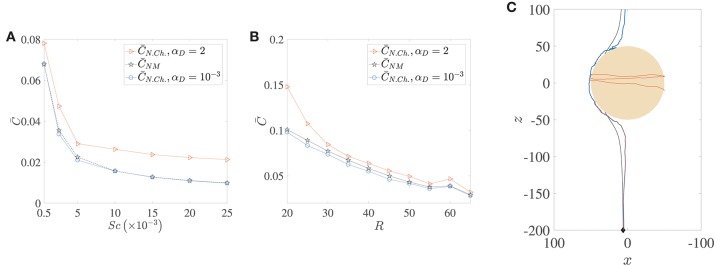
A comparison of the average nutrient exposure, $\bar{C}$, between motile but non-chemotactic bacteria and non-motile bacteria, as a function of **(A)** the nutrient diffusivity, with dimensionless aggregate radius *R* = 45, and **(B)** the (dimensionless) radius of the marine snow particle, *R*, with Schmidt number *Sc* = 25000. **(C)** Sample trajectories for the three cases whose nutrient exposures are plotted in **(B)**, *R* = 50, with correspondence based on line colors. The blue and brown trajectories are indistinguishable until they near the aggregate, and the latter gets trapped. Notice the “smoothness” of the non-motile trajectory (black) vs. that of the non-chemotactic trajectory of bacterium with weak hydrodynamic interaction (blue). The nutrient exposure for motile, non-chemotactic bacteria has been evaluated for both strong (α_*D*_ = 2) and weak ($\alpha_{D}=10^{-3}$) hydrodynamic interactions. The other parameters are: *K*_Δρ_ = 0.0109, τ^*^ = 1.

The locomotion of motile bacteria–both chemotactic and non-chemotactic–was discussed in detail in Sections 2.1, 2.2 and the Appendix. In comparison, the motion of non-motile bacteria in the ocean is fairly simple: they just act as passive tracers being carried by the fluid flow. It has been shown in the past that the nutrient exposure is more or less the same for motile, non-chemotactic bacteria when compared to non-motile bacteria, over a wide range of initial conditions and Péclet numbers (Jackson, 1989). This is indeed accurate if hydrodynamic interactions are negligible, as seen in the comparison between the pentagrams and circles in Figure 9A. In fact, for our simulations, ${\bar{C}}_{NM}$ was slightly larger than ${\bar{C}}_{N.Ch.}$ for a wide range of marine snow radii, when hydrodynamic interactions were particularly weak (see Figure 9B). But stronger hydrodynamic interactions greatly improve the nutrient exposure for the non-chemotactic bacteria with the percentage increase

(8) $$\begin{array}{l} A_{C2}=\frac{\left({\bar{C}}_{N.Ch.}-{\bar{C}}_{NM}\right)}{{\bar{C}}_{NM}}\times 100, \end{array}$$

being even greater than 100% (i.e., more than two-fold increase) for the case of the scarcest nutrient availability (*Sc* = 2.5 × 10^4^, *Pe* = 5000). The amplification is a little less drastic as a function of aggregate size though, with enhanced hydrodynamic interactions enabling the non-chemotactic bacteria to profit by *A*_*C*2_ ≈ 40% for the highest aggregate size, and by ≈ 20% for the lowest. This is a significant contribution and hints at potential motility induced advantage, irrespective of the chemotactic nature of marine microorganisms. Needless to say, the (motile) chemotactic bacteria are always at an advantage with respect to the non-motile bacteria and therefore we do not discuss their comparison in this section.

## 4. Conclusion

In this paper, we investigated the combined influence of hydrodynamics and chemotaxis on the colonization of sinking nutrient sources by marine bacteria. We first developed and simulated a comprehensive mathematical model incorporating bacterial swimming as influenced by: (i) fluid flow, and, (ii) chemotaxis toward the nutrient-rich regions surrounding and trailing a sedimenting marine snow aggregate. In addition to swimming with respect to the ambient fluid, bacteria are rotated and translated due to hydrodynamic interactions with nearby surfaces, such as the sinking aggregate in our case. These interactions, if sufficiently strong, can passively trap bacteria that stray too close to the aggregate and thus play a major role in enhancing a bacterium's stay in the nutrient hot-spots in marine ecosystems. We quantified the critical value of aggregate radius above which oncoming bacteria are trapped, and its dependence on the aggregate's excess density and the bacterial dipole strength (dimensionless propulsive force exerted by a bacterium on the fluid). The critical trapping radius was lowest for the smallest excess densities and largest dipole strengths. We note however, that the analysis of the critical trapping radius was carried out in absence of noise/rotational diffusion of the bacterium. In presence of noise, the bacterium's interaction with the aggregate was quantified via a trapping fraction, $F_{trap}$. This is a measure of the likelihood of a bacterium being captured onto the aggregate surface, when its orientation is affected by thermal and/or athermal fluctuations. We showed that the phenomenon of hydrodynamic trapping is robust to noise, and discussed how factors such as the aggregate's radius and excess density can affect the trapping fraction.

Even though the attractive nature of the hydrodynamic interactions is restricted to within a few body-lengths from the aggregate, we showed that it can drastically alter a marine bacterium's nutrient exposure. For example, chemotactic bacteria with higher dipole strengths had ≈ 40% greater nutrient exposure, as compared to chemotactic bacteria with relatively lower dipole strengths. A quintessential scenario when such large amplifications could occur is the bacterial encounter of sinking phytoplankton cells (*d* ≈ 100μm) exuding low molecular weight glycolates (Bowen et al., 1993; Jackson, 2012). Interestingly, this advantage is not restricted to chemotactic bacteria alone. Due to the purely hydrodynamic nature of the trapping phenomenon, any motile bacteria lying in an aggregate's swept volume can potentially get trapped onto its surface. Hydrodynamics therefore, can yield substantial nutritional benefit even to *non-chemotactic, but motile* bacteria, when compared to the non-motile bacteria. These benefits depend on a variety of environmental conditions and biological parameters, like the size of the marine snow, the molecular diffusivity of the nutrient under consideration and the average run-length of bacterial species. We systematically studied these variations and provided an explanation for the obtained trends based on the influence of hydrodynamic and/or chemotactic effects. In particular, we demonstrated that hydrodynamics becomes progressively more important as the bulk nutrient availability–quantified by a concentration boundary layer thickness–declines, especially for non-chemotactic bacteria. This is particularly significant because the diffusion coefficients of the nutrients consumed by marine bacteria vary over a few orders of magnitude (10^−8^ cm^2^/s < *D*_*C*_ < 10^−5^ cm^2^/s). Our results thus suggest that bacteria can accrue substantial nutritional gains due to motility, particularly when foraging for high molecular weight (thus low diffusion coefficient) solutes which form a major part of available dissolved organic matter in oceans (Amon and Benner, 1994). In contrast, we showed that larger aggregates (marine snow particles with radii greater than 1 mm) proved too fast for the bacteria to get trapped onto, thus diminishing the role played by hydrodynamics in those regimes. An implication of the nutrient source's speed being very high (in comparison to the bacterial swimming speed) is that rising crude oil drops are not amenable to hydrodynamic trapping. Their “excess” densities are quite large (Δρ ≈ −0.15 g/cm^3^), thus preventing hydrodynamic trapping to occur at all, even for the small drops of diameter ≈ 1 mm. We performed simulations like those discussed before, for rising oil drops and found that the amplification is practically non-existent, irrespective of bacteria being chemotactic or non-chemotactic. Therefore, bacteria must attach onto the rising oil drops via interfacial phenomena other than near-surface hydrodynamics, possibly via adsorption after a random encounter (Vaccari et al., 2017; Dewangan and Conrad, 2018; McLay et al., 2018). However, surfactant addition breaks down larger oil drops into droplets ranging from 20-60 μm in diameter (Atlas and Hazen, 2011), which are almost neutrally buoyant and get trapped in sub-surface hydrocarbon plumes (Camilli et al., 2010; Ryerson et al., 2012) or pycnoclines (Paris et al., 2012). In these cases, hydrodynamics does affect the accumulation of bacteria around oil drops. Specifically, hydrodynamics enables surfactant-laden drops to trap bacteria more effectively than surfactant-free drops (Desai et al., 2017), and strong hydrodynamic interactions increase the bacterial colonization of oil drops by ≈ 60% (in comparison to weak hydrodynamic interactions; Desai and Ardekani, 2018).

Our study reveals a passive, non-trivial mechanism that enables marine bacteria to reside on, and populate, moving nutrient sources in the ocean. A key insight is the generality of the hydrodynamic aspects of the results, which do not depend heavily on the details of the bacteria involved. This enables one to use the derivations presented here in combination with different active behaviors–chemotactic or otherwise–to investigate a variety of phenomena involving motile bacteria in fluid flows past nutrient sources. The present work reveals some intricacies of the initial stages of microbial colonization of nutrient sources, and extensions can be developed over the framework presented here. If the rate of aggregate consumption is slow, then our analysis can be extended to the case of time-varying aggregate size by simply replacing the constant **F**_*ext*_ by some time-dependent **F**_*ext*_(*t*). The number of bacteria in the simulation would have to be continuously updated over such longer time scales, with possible alterations to their surface motility, e.g., a change from near-surface swimming/swarming to surface twitching/gliding (Mazza, 2016). The analysis can also be extended to elongated bacteria (as opposed to spherical cells considered in this paper) to explore the effects of cell shape on nutrient colonization. Other details in the bacterium's intrinsic motility–like chemokinesis, near-surface tumbling–are also easy to add in the present study, given the availability of experimental data (Molaei et al., 2014; Molaei and Sheng, 2016; Son et al., 2016). In this way, the model described can be extended, in conjunction with observations, to incorporate finer details like evolution of microbial demographics based on surface accumulation and substrate consumption. We thus envision rich applications of this study toward analyzing complex processes involving close association of fluid flow and active motility, e.g., bioremediation, microswimmer sorting/isolation, predator-prey interactions at the micron scale.

## Data Availability

Data are publicly available through the Gulf of Mexico Research Initiative Information & Data Cooperative (GRIIDC) at https://data.gulfresearchinitiative.org (doi: 10.7266/n7-1wdy-1e57).

## Author Contributions

ND and AA designed the research. ND and VS performed the research. ND and AA analyzed the data. ND and AA wrote the manuscript.

### Conflict of Interest Statement

The authors declare that the research was conducted in the absence of any commercial or financial relationships that could be construed as a potential conflict of interest.
