# Supplementary material for: Hydrodynamic Interaction Enhances Colonization of Sinking Nutrient Sources by Motile Microorganisms
Source: Front Microbiol. 2019 Mar 12;10:289. doi: 10.3389/fmicb.2019.00289 (PMC6422982; doi:10.3389/fmicb.2019.00289)
Supplement: Supplementary file 1 [file Data_Sheet_1.pdf]

Hydrodynamic Interaction Enhances Colonization of Sinking Nutrient Sources by Motile Microorganisms

Nikhil Desai[1], **Vaseem A. Shaik**[1] **and Arezoo M. Ardekani**[1,*]

[1] *School of Mechanical Engineering, Purdue University, West Lafayette, Indiana, 47907, USA*

Correspondence*:
Corresponding Author
ardekani@purdue.edu

APPENDIX

Appendix A: Equations governing fluid flow

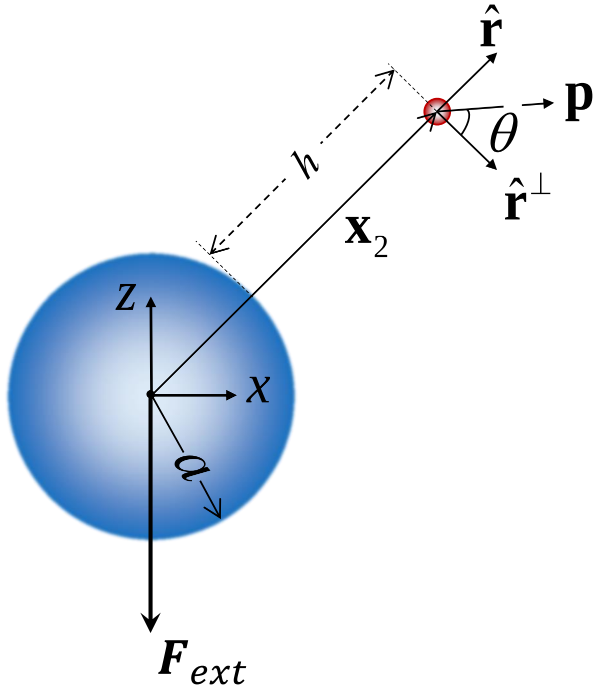

Figure 1. Schematic depicting the coordinate systems, the vectors and the symbols used to describe the linear and angular velocities of the aggregate and the bacterium. The simulations are performed in the coordinate system xyz, which is fixed to the center of the marine snow particle, with the y axis pointing into the plane of the figure. The coordinate system defined by the orthogonal vectors $\hat{\mathbf{r}}$, $\hat{\mathbf{r}}^{\perp}$ and $\hat{\mathbf{r}}^{\perp} \times \hat{\mathbf{r}}$ can translate and rotate with respect to xyz. Note that h is the dimensionless separation of the bacterium (assumed to be spherical in our analysis) from the aggregate and θ is the in-plane angle, i.e., the angle between the bacterium's orientation $\mathbf{p}$ and the unit vector $\hat{\mathbf{r}}^{\perp}$.

The fluid velocity, $\mathbf{v}$, and pressure, P, are governed by the conservation of mass,

$$\nabla \cdot \mathbf{v} = 0, \tag{1}$$

and the conservation of momentum under negligible inertia (Stokes equations),

$$-\nabla P + \mu \nabla^2 \mathbf{v} + \mathbf{F}_D = 0, \tag{2}$$

where, μ is the dynamic viscosity of the suspending fluid. $\mathbf{F}_D$ is the contribution of the bacterium to the fluid flow, which is well approximated by a force dipole. Mathematically it can be represented as a difference between two point forces separated by a distance b (which is the characteristic size of the microorganism):

$$\mathbf{F}_D = f_d \mathbf{p} \delta \left\{ \mathbf{x} - (\mathbf{x}_2 + b\mathbf{p}/2) \right\} - f_d \mathbf{p} \delta \left\{ \mathbf{x} - (\mathbf{x}_2 - b\mathbf{p}/2) \right\}, \tag{3}$$

where δ is the three dimensional Dirac delta function, $\mathbf{x}_2$ is the location of the bacterium's center and $\mathbf{p}$ is the bacterium's orientation. More formally, a Taylor series expansion of the two terms in eqn. 3 yields the force dipole to be the gradient of a point force along $\mathbf{p}$, taken in the direction of $\mathbf{p}$ itself,

$$\mathbf{F}_D = F_D \left(\mathbf{p} \cdot \nabla \right) \left\{ \mathbf{p} \delta \left(\mathbf{x} - \mathbf{x}_2 \right) \right\}, \tag{4}$$

the quantity $F_D \approx f_d b$ is called the dipole strength of the microorganism.

Eqns. 1 and 2 need to be solved subject to the boundary condition

$$\mathbf{v}\left(\mathbf{x}_s\right) = \mathbf{U}_p + \mathbf{\Omega}_p \times \mathbf{x}_s, \tag{5}$$

where $\mathbf{x}_s$ corresponds to the surface of the marine snow particle, $\mathbf{U}_p$ is the velocity with which the particle is sinking and $\mathbf{\Omega}_p$ is its angular velocity. Note that if the bacterium is not present (i.e., $F_D = 0$), then $\mathbf{\Omega}_p = \mathbf{0}$, and $\mathbf{U}_p$ is given by the Stokes settling velocity $\mathbf{F}_{ext}/(6\pi\mu a)$. But the presence of the bacterium, and concomitant hydrodynamic interactions, mean that $\mathbf{U}_p$ and $\mathbf{\Omega}_p$ are not known *a priori*. So, we utilize a well-known technique called the 'method of reflections' (Kim and Karrila, 1991) to obtain $\mathbf{u}_{HI}$, $\mathbf{\Omega}_{HI}$, $\mathbf{U}_p$ and $\mathbf{\Omega}_p$. The resulting expressions are provided in Appendix B [eqns. 6 to 13]. It is very important to note the dependence of these expressions on the quantity α_D, which is the dimensionless dipole strength.

Appendix B: Expressions for hydrodynamically induced translational and angular velocities

The relevant expressions on the right-hand-side of eqn. 1 in the main manuscript are obtained after the application of the 'method of reflections' and Faxén laws for the marine snow particle and the bacterium (Kim and Karrila, 1991). They are represented in terms of the unit vectors $\hat{\mathbf{r}}$ and $\hat{\mathbf{r}}^{\perp}$. $\hat{\mathbf{r}}$ is directed along the line joining the center of the aggregate to the bacterium, and $\hat{\mathbf{r}}^{\perp}$ is perpendicular to $\hat{\mathbf{r}}$, as shown in Fig. 1. The coordinate system defined by the unit vectors $\hat{\mathbf{r}}$, $\hat{\mathbf{r}}^{\perp}$ and $\hat{\mathbf{r}}^{\perp} \times \hat{\mathbf{r}}$ can rotate and translate with respect to the body-fixed coordinate system xyz. The expression for $\mathbf{u}_{HI}$ is:

$$\frac{\mathbf{u}_{HI}}{V_s} = \mathbf{u}_{HI,1} + \mathbf{u}_{HI,2} + \mathbf{u}_{HI,3}, \tag{6}$$

with,

$$\mathbf{u}_{HI,1} = \frac{3}{4}\frac{R}{R+h}\left[\bar{\mathbf{F}}_{ext}\left\{1 + \frac{1}{3}\left(\frac{R}{R+h}\right)^2\right\} + \left(\bar{\mathbf{F}}_{ext} \cdot \hat{\mathbf{r}}\right)\hat{\mathbf{r}}\left\{1 - \left(\frac{R}{R+h}\right)^2\right\}\right], \tag{7}$$

$$\mathbf{u}_{HI,2} = -\frac{3R\alpha_D\left(1-3\sin^2\theta\right)(R+h)}{2h^2(2R+h)^2}\hat{\mathbf{r}} + \frac{3R^3\alpha_D\left(2R^2+6Rh+3h^2\right)\sin 2\theta}{4h^2(2R+h)^2(R+h)^3}\hat{\mathbf{r}}^{\perp}, \tag{8}$$

and

$$\mathbf{u}_{HI,3} = \frac{3}{4}\frac{\alpha_D R}{(R+h)^3}\left[\begin{array}{c} \left(\frac{R}{R+h}\right)^2\left\{3+\frac{1}{3}\left(\frac{R}{R+h}\right)^2\right\}\sin(2\theta)\,\hat{\mathbf{r}}^{\perp} \\ -2\left(1-3\sin^2\theta\right)\left\{1-\frac{4}{3}\left(\frac{R}{R+h}\right)^2+\frac{1}{3}\left(\frac{R}{R+h}\right)^4\right\}\hat{\mathbf{r}} \end{array}\right]. \tag{9}$$

The expression for $\mathbf{\Omega}_{HI}$ is:

$$\frac{\mathbf{\Omega}_{HI}}{V_s/b} = \mathbf{\Omega}_{HI,1} + \mathbf{\Omega}_{HI,2} + \mathbf{\Omega}_{HI,3}, \tag{10}$$

with

$$\mathbf{\Omega}_{HI,1} = \frac{3}{4}\frac{R}{(R+h)^2}\left(\bar{\mathbf{F}}_{ext}\times\hat{\mathbf{r}}\right), \tag{11}$$

$$\mathbf{\Omega}_{HI,2} = -\frac{3R^3\alpha_D\left(2R^2+6Rh+3h^2\right)\sin 2\theta}{4h^3(2R+h)^3(R+h)^2}\left(\hat{\mathbf{r}}^{\perp}\times\hat{\mathbf{r}}\right), \tag{12}$$

and

$$\mathbf{\Omega}_{HI,3} = \frac{3}{2}\alpha_D\left\{\frac{R}{(R+h)^2}\right\}^3\sin(2\theta)\left(\hat{\mathbf{r}}^{\perp}\times\hat{\mathbf{r}}\right). \tag{13}$$

All the terms in the right-hand-side of eqns. 7 to 9, 11 to 13 are dimensionless, and are given by:

$$R = \frac{a}{b},\ h = \frac{(|\mathbf{x}_2| - a)}{b},\ \alpha_D = \frac{F_D}{8\pi\mu b^2 V_s},\ \bar{\mathbf{F}}_{ext} = \frac{\mathbf{F}_{ext}}{6\pi\mu a V_s}. \tag{14}$$

In the above equations, h is the dimensionless separation of the microorganism from the surface of the marine snow, and the θ is the angle between the bacterium's orientation $\mathbf{p}$ and the unit vector $\hat{\mathbf{r}}^{\perp}$ (see Fig. 1). The terms given by eqns. 8, 9, 12 and 13 arise due to the flow generated by the bacterium (hence the contribution of α_D); while those given by eqns. 7 and 11 arise due to the fluid flow cause by the settling sphere. Although the latter terms dominate when $|\mathbf{x}_2|$ is large, the former terms can become significant as $|\mathbf{x}_2|$ reduces, i.e., as the bacterium approaches the sphere/aggregate.

The velocity of the settling marine snow particle, as altered by the presence of the microorganism, is given by:

$$\frac{\mathbf{U}_p}{V_s} = \frac{\mathbf{F}_{ext}}{6\pi\mu a V_s} + \frac{\alpha_D}{|\mathbf{x_2}|^2/b^2}\left[\frac{a^2}{|\mathbf{x_2}|^2}\sin(2\theta)\,\hat{\mathbf{r}}^{\perp} - \left(1-3\sin^2\theta\right)\left\{1-\frac{a^2}{|\mathbf{x_2}|^2}\right\}\hat{\mathbf{r}}\right], \tag{15}$$

and the angular velocity induced due to hydrodynamic interactions is:

$$\frac{\mathbf{\Omega}_p}{V_s/b} = -\frac{3\alpha_D}{2\left(|\mathbf{x_2}|^3/b^3\right)}\sin(2\theta)\left(\hat{\mathbf{r}}^{\perp}\times\hat{\mathbf{r}}\right). \tag{16}$$

The second term on the right hand side of eqn. 15 is the correction to the Stokes settling speed due to the presence of the microorganism. The quantity $|\mathbf{x}_2| \geq a$, and thus the effect of the microorganism on the marine snow's settling speed (and consequently, on the fluid flow and nutrient distribution) can be

neglected if

$$\frac{|\mathbf{F}_{ext}|}{6\pi\mu a V_s} \gg \frac{\alpha_D}{R^2}, \tag{17}$$

where, recall from eqn. 14 that $R = a/b$. Now, considering $\mathbf{F}_{ext} = 4/3\pi a^3 \Delta\rho \mathbf{g}$ (the marine snow particle is sinking under gravity), we have,

$$\frac{16\pi}{9}\frac{\Delta\rho g a^4}{F_D} \gg 1, \tag{18}$$

which is typically satisfied for the parameter values listed in Table 1 in the main manuscript. Similarly, the effect of rotation of the marine snow on the nutrient transport can be neglected based on the inequality $|\mathbf{U}_p| >> |\mathbf{\Omega}_p \times a\hat{\mathbf{r}}|$, or,

$$\frac{|\mathbf{F}_{ext}|}{6\pi\mu a V_s} \gg \frac{3\alpha_D}{2R^2}, \tag{19}$$

which yields,

$$\frac{32\pi}{27}\frac{\Delta\rho g a^4}{F_D} \gg 1. \tag{20}$$

Eqns. 18 and 20 allow us to neglect the effect of aggregate-bacterium hydrodynamic interactions on the nutrient transport (eqn. 2 in the main manuscript). In addition, the Reynolds number corresponding to the marine snow aggregates considered in this work, $Re_{ms} << 1$; hence, we use,

$$\mathbf{v}_{St} = -\mathbf{U}_{p,0} + \left(\frac{3a}{4r} + \frac{a^3}{4r^3}\right)\mathbf{U}_{p,0} + \left(\frac{3a}{4r^3} - \frac{3a^3}{4r^5}\right)\mathbf{x}\left(\mathbf{U}_{p,0}\cdot\mathbf{x}\right), \tag{21}$$

in eqn. 2 in the main manuscript. Eqn. 21 is the flow field due to a sphere being acted upon by an external force $\mathbf{F}_{ext}$, in the regime of negligible inertia. In eqn. 21, $r = |\mathbf{x}|$ and $\mathbf{U}_{p,0} = \mathbf{F}_{ext}/(6\pi\mu a)$ is the velocity of a sphere of radius a in presence of an external force $\mathbf{F}_{ext}$ and negligible inertia, obtained by substituting $\alpha_D = 0$ in eqn. 15, hence the sub-script '0'.

Appendix C: Simulation details

Nutrient concentration

We solved eqn. 2 in the main manuscript using a finite element method, and validated our code by comparing the value of the Sherwood number (as a function of Péclet number) with analytical and numerical predictions (see Fig. 2 in the Appendix).

Bacteria trajectories

We simulate the mathematical model described in sections 2.1 and 2.2 of the main manuscript, for a system containing $N_b = 1000$ non-interacting bacteria, placed uniformly within a disk of radius $2a$ at a vertical separation $5a$ below the sinking marine snow particle. The simulations are run until either the bacteria are at separations greater than $r_{lim} = 50a$ from the center of the marine snow, or the maximum simulation time is reached. As we are only considering bacteria upstream from the settling marine snow and from within the disc, we are actually neglecting any bacteria that could drift in from above the marine snow, or from 'the side'. However, as long as the aggregate settles at a rate much faster than the bacterial swimming speed, we can safely neglect the drifting in of any bacteria from above the marine snow. Also, the time taken by a bacterium to diffuse in from the sides via a random walk is $t_d \sim a^2/(V_s^2\tau_0/6)$. Therefore, as long as the aggregate falls a distance greater than a in this time, i.e., as long as $U_p t_d >> a$, the diffusion

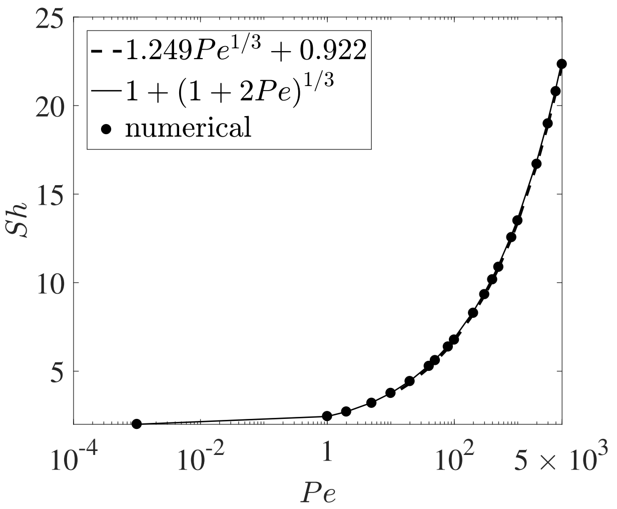

Figure 2. The Sherwood number—a dimensionless measure of the mass transfer rate at the sphere surface—as a function of the Péclet number evaluated using numerical simulations and compared against analytical predictions for $Pe >> 1$ [dashed line, by (Acrivos and Goddard, 1965)] and numerical predictions valid for all Péclet numbers [solid line, by (Clift et al., 1978)].

of bacteria from the sides can also be neglected. In our case, the minimum value of the ratio $U_p t_d / a$ is ≈ 50, and so bacteria diffusing in from the sides would not get ample time to locate the nutrient source (Kiørboe and Jackson, 2001).

We emphasize that the value of the threshold bacteria-aggregate separation above which the simulations are stopped (i.e., r_{lim}), has a minor quantitative effect on the final results, with all our descriptions of the possible qualitative behaviors staying the same. Fig. 3 shows that increasing r_{lim} by a factor of two is seen to affect the $\bar{C}$ values of chemotactic bacteria most acutely—by almost 10%—for the case of strongest hydrodynamic interaction. The values of $\bar{C}$ for chemotactic bacteria with weak hydrodynamic interaction, and those for non-chemotactic bacteria do not change appreciably. The maximum change in the values of A_C for chemotactic bacteria is by only 3%, suggesting that our predictions are robust against changes to r_{lim}. It can also be seen that using larger number of bacteria, say, $N_b = 5000$, does not have a very significant quantitative effect on our results (see Fig. 6B in Section 3.2 of the main manuscript).

We march the equations governing $\mathbf{x}_2(t)$ and $\mathbf{p}(t)$ in time using an explicit Euler method, and track the position and the nutrient concentration to which the bacteria are exposed. We compute DC/Dt for the bacteria at each time-step Δt, and effect a tumble if the quantity $\Delta t/\tau > \mathscr{R}$, where τ is given by eqn. 3 in the main manuscript, and $\mathscr{R}$ is a uniformly chosen random number from [0,1] (Frymier et al., 1994; Duffy et al., 1995). The tumble is implemented by changing the bacterium's orientation from $\mathbf{p}$ to $\mathbf{p}'$, with there being no correlation between $\mathbf{p}$ and $\mathbf{p}'$ (isotropic tumbles). The implementation of correlated tumbles, including the 'run-reverse-flick' strategy, is straightforward in that the angle between $\mathbf{p}'$ and $\mathbf{p}$ must be chosen from a prescribed, non-uniform distribution. We also assume that the tumbles are instantaneous. It is easy to see that whenever $DC/Dt > 0$, the tumbling probability $\Delta t/\tau$ is smaller than the unbiased tumbling probability $\Delta t/\tau_0$, and so a bacterium moving up a nutrient gradient is less likely to veer off in a different direction.

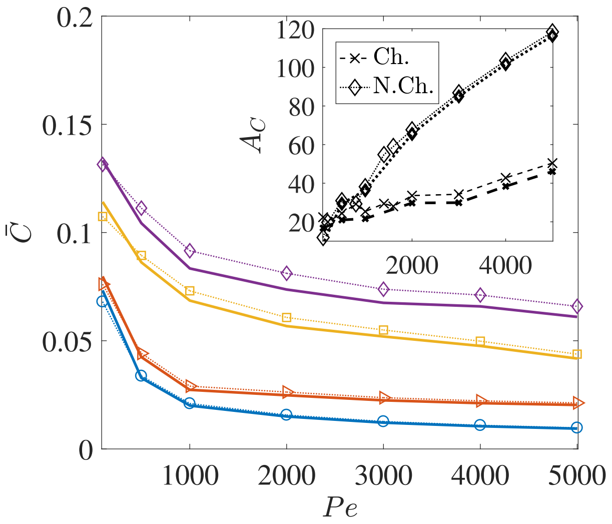

Figure 3. The variation in the average nutrient exposure, $\bar{C}$, for chemotactic and non-chemotactic bacteria, with $r_{lim} = 25$ (thick lines) and $r_{lim} = 50$ (thin lines). The legends in the main figure are as follows: $\Diamond$—chemotactic, $\alpha_D = 2$; $\Box$—chemotactic, $\alpha_D = 0.1$; $\triangleright$—non-chemotactic, $\alpha_D = 2$; $\bigcirc$—non-chemotactic, $\alpha_D = 0.1$. Note how r_{lim} affects $\bar{C}$ the most for chemotactic bacteria with $\alpha_D = 2$: this is because the bacteria that get hydrodynamically trapped contribute the same amount to the overall mean nutrient exposure irrespective of the value of r_{lim}.

REFERENCES

Acrivos, A. and Goddard, J. D. (1965). Asymptotic expansions for laminar forced-convection heat and mass transfer. *Journal of Fluid Mechanics* 23, 273. doi:10.1017/S0022112065001350

Clift, R., Grace, J., and Weber, M. (1978). *Bubbles, drops and particles* (New York: Academic Press)

Duffy, K., Cummings, P., and Ford, R. (1995). Random walk calculations for bacterial migration in porous media. *Biophysical Journal* 68, 800–806. doi:10.1016/S0006-3495(95)80256-0

Frymier, P. D., Ford, R. M., and Cummings, P. T. (1994). Analysis of bacterial migration: I. Numerical solution of balance equation. *AIChE Journal* 40, 704–715. doi:10.1002/aic.690400413

Kim, S. and Karrila, S. (1991). *Microhydrodynamics: Principles and Selected Applications* (Boston: Butterworth- Heinemann)

Kiørboe, T. and Jackson, G. A. (2001). Marine snow, organic solute plumes, and optimal chemosensory behavior of bacteria. *Limnology and Oceanography* 46, 1309–1318. doi:10.4319/lo.2001.46.6.1309
